# Copper salt impregnated biomass-derived microporous carbon for hydrogen storage

**DOI:** 10.1039/d6lf00048g

**Published:** 2026-05-15

**Authors:** Lila A. M. Mahmoud, Charles D. Brewster, Lui Skytree, Misbah Khan, Sebastien Rochat, Valeska P. Ting, David J. Fermin, Jemma L. Rowlandson, Sanjit Nayak

**Affiliations:** a School of Chemistry, Cantocks Close, University of Bristol Bristol BS8 1TS UK; b School of Civil, Aerospace and Design Engineering, University of Bristol Bristol BS8 1TR UK s.nayak@bristol.ac.uk; c School of Chemistry and Biosciences, University of Bradford BD7 1DP UK; d Bristol Composites Institute, University of Bristol Bristol BS8 1TR UK; e School of Electrical, Electronic and Mechanical Engineering, University of Bristol Bristol BS8 1TR UK J.Rowlandson@bristol.ac.uk; f Research School of Chemistry, Australian National University Canberra ACT 2601 Australia; g School of Engineering Mathematics and Technology, University of Bristol Bristol BS8 1TW UK

## Abstract

Biomass derived carbon is a promising class of sustainable porous materials for hydrogen storage. The natural vascular structure of plant biomass can potentially facilitate uptake and even dispersion of metal ions from aqueous solution, enabling activation during carbonisation. To test this hypothesis, we studied a one-step pre-activation approach for synthesis of microporous carbon using copper(ii) ions and kale plant stems as a model vascular biomass precursor. Thermogravimetric analysis (TGA) shows systemic changes in the mass loss profiles and increased residual inorganic content with higher CuCl_2_ loading. Surface area analyses, Raman spectroscopy and transmission emission microscopy (TEM) demonstrate activation and localised structural ordering by the copper salt up to an optimal threshold. Nitrogen adsorption measurements confirmed the formation of ultramicroporous activated carbon, with a BET surface area of 385 m^2^ g^−1^ and total hydrogen uptake of 1.78 wt% at 50 bar and 77 K. An increase in porosity was observed with increasing metal-ion concentration. The findings highlight the pre-activation of vascular biomass precursors using metal ions as an effective strategy to create porous carbons, and the dual role of CuCl_2_ in tailoring carbon structure and porosity, offering a combined sustainable route to prepare high-performance hydrogen storage materials from plant-based precursors.

## Abstract 2

Biomass derived carbon as a promising class of sustainable porous materials for hydrogen storage.

## Graphical abstract

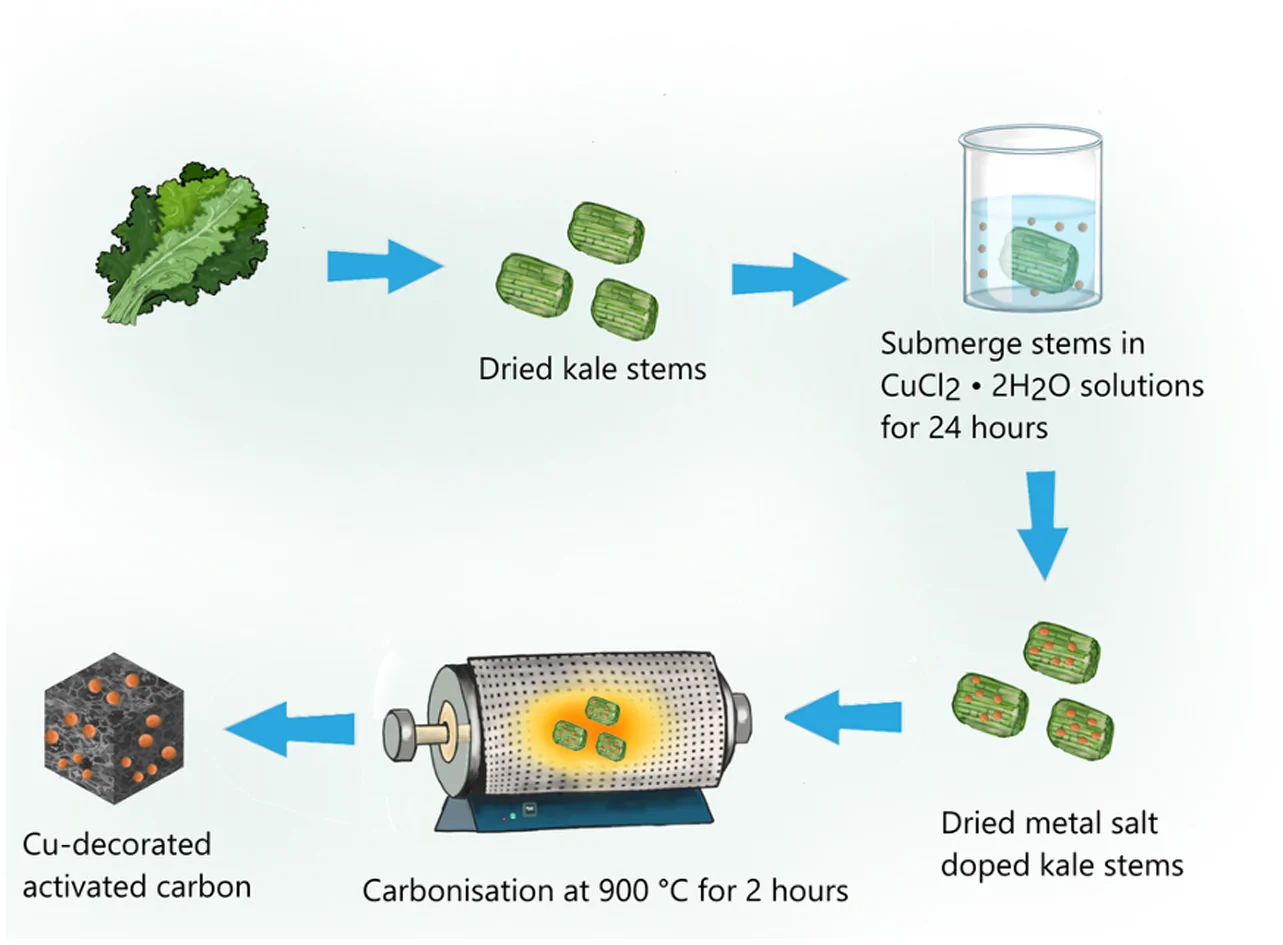

## 1. Introduction

A crucial challenge of the hydrogen economy is linked to its efficient storage and transportation.^1^ Despite having high gravimetric energy density, hydrogen is difficult to store and transport due to its low volumetric density.^2^ Increasing the volumetric density of hydrogen is essential for efficient storage. However, physical storage methods require very high pressures or cryogenic temperatures, which raises significant cost and safety concerns.^3^ Other hydrogen storage solutions include materials-based approaches, where hydrogen is stored into materials through chemical or physical interactions. Physical interactions are referred to as physisorption, where hydrogen is weakly bound to the surface through van der Waals forces, and properties like high surface area and accessible porosity play critical roles for effective storage. Porous materials such as metal–organic frameworks (MOFs), covalent organic frameworks (COFs), polymers of intrinsic microporosity (PIMs), and porous carbon have been explored for the physisorption of hydrogen, owing to their high surface area.^4–7^

Activated carbons constitute another promising class of materials for hydrogen storage applications with high surface area ranging from 500 to 3000 m^2^ g^−1^. Porous carbon materials can be classified into different categories, including activated carbon, carbon nanotubes, fullerenes, graphene-derived materials, and template derived and carbide derived carbons. Activated carbons have the advantage of low cost and scalability, whereas graphene derived and template derived carbons provide more controlled porosity through tailored architectures *via* complex synthesis methods. These different types of carbons have different preparation methods, which directly influence pore structure, surface chemistry and subsequently, hydrogen storage capacity. Graphene-based, and silica or zeolite-derived templated carbons can offer highly narrowed pores that can achieve high gravimetric hydrogen storage capacity. Graphene-based carbon materials have been reported to achieve adsorption capacities approaching 7 wt% under cryogenic conditions. This is due to their reported theoretical high surface area and tuneable interlayer spacing. Similarly, template derived carbons using soft or hard templates achieve narrowed pore structures and surface areas exceeding 3000 m^2^ g^−1^ (see Table S1), resulting in enhanced hydrogen uptake.^7–9^ However, such materials rely on multistep synthesis routes, expensive templates, and aggressive etching processes, which limits their scalability.^10^ As summarised in Table S1, biomass-derived activated carbons can exhibit surface areas in the range of 1500–3000 m^2^ g^−1^ and hydrogen storage capacities ranging from 1.5 to 3.5 wt% at 1 bar and 77 K. While surface area is an important parameter, it has been shown in literature that the hydrogen uptake strongly correlates with the presence of ultramicropores (<0.7 nm), which provide enhanced adsorption due to overlapping potential fields. Consequently, materials with similar surface areas can exhibit different hydrogen storage capacities depending on their pore size distribution.

**Scheme 1:**
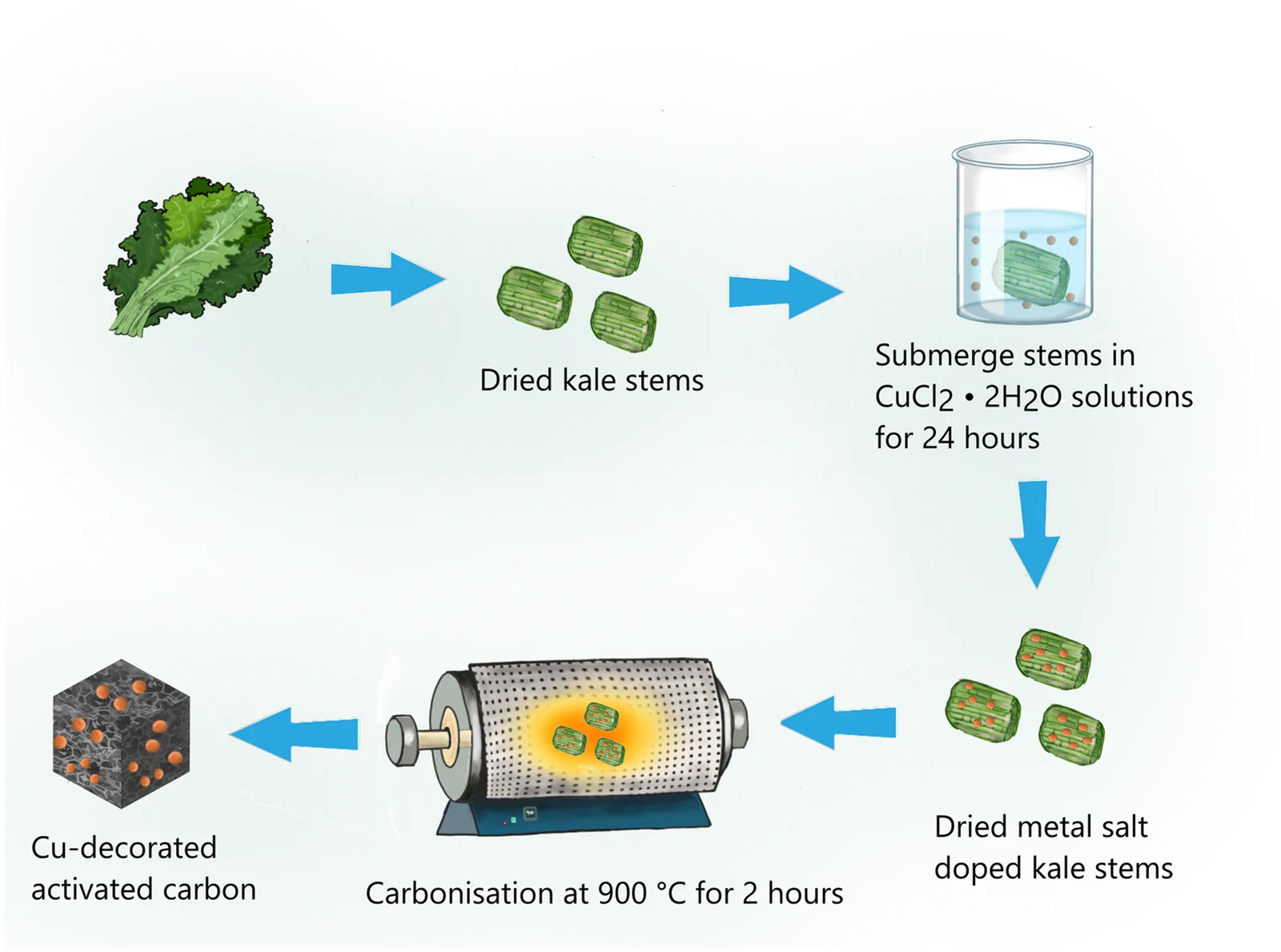
Schematic showing the impregnation of biomass with metal ions followed by carbonisation to generate metal salt activated biomass derived carbon.

For hierarchical porous carbons on the other hand, activated carbons can be synthesised from various carbon-rich precursors through a carbonisation process, often followed by a second step called activation to develop further porosity.^11^ Activation is often performed by physical or chemical methods.^12^ Physical activation involves the use of oxidizing gases at high temperatures,^13,14^ while chemical activation involves the use of chemical agents that interact with the carbon precursor during carbonisation.^15^ Traditionally, chemical activation involves the use of activating agents such as ZnCl_2,_ KOH, K_2_CO_3_, or H_3_PO_4_. These agents facilitate etching of the carbon matrix at high temperature, which removes carbon atoms and can generate porosity. However, effective traditional activation adds an additional step which is often energy intensive and carried out at high temperature.^16^ In addition, KOH can cause pore collapse due to over-etching, resulting in lower carbon yield, and loss of porosity.^17^ Hence, given the drawbacks associated with additional activation step, there is a need to explore alternative activation methods.^18^ Recent studies have shown several advantages of using transition metal based activation agents. Transition metals possess multiple accessible oxidation states, enabling redox reactions that promote carbon gasification and pore formation. Such transition metal based activating agents can also provide enhanced control of pore sizes.^17,19–23^ In addition, transition metals can catalyse the graphitisation of disordered carbon, leading to the formation of ordered sp^2^-hybridised graphitic nanostructures.^24^ This simultaneous activation and graphitisation requires further understanding and refining to better control synthesis methods to increase surface area and subsequently, optimise materials properties (Scheme 1).^25^

Among different kinds of precursors that have been studied for preparing activated carbon, biomass precursors can offer unique advantages such as being abundant, renewable, and structurally diverse.^26^ In addition, plant-derived biomass sources possess inherent vascular architectures which can enable uniform dispersion of activating agents besides serving as natural templates for porous carbon formation. These templated structures can be preserved after carbonisation, and can contribute to multi-scale porosity. In addition, the introduction of activation agents within these organised frameworks can result in the formation of hierarchically porous carbon material.^27^

Transition metal salts have been widely investigated for porous carbon synthesis. For example, Zn(ii)-based salts are among the most studied activating agents for various applications, particularly supercapacitors and electrode fabrication.^28^ Transition metal salts of Ni(ii), Fe(iii), and Cu(ii) ions have also been explored using a range of biomass precursors, such as eucalyptus sawdust, cotton waste, and corn cobs, yielding porous carbons tailored for applications including electrochemical energy storage and heavy metal removal.^29–32^

Although several studies have explored CO_2_ capture using metal salt activated carbon,^33,34^ the application of transition metal salts as activation agents and optimising tool for hydrogen storage is relatively unexplored. This is especially notable given that some metal salts are highly effective at tuning microporosity and pore dimension, which play critical roles for hydrogen adsorption.^35^

In this work, we report the synthesis of hierarchical porous carbons, derived from kale plant vegetable stems as biomass precursor. This precursor was selected as a model system with its vascular structure that can facilitate uniform dispersion of metal salts *via* capillary forces. The biomass was impregnated with different concentrations of CuCl_2_ followed by a one-step simultaneous carbonisation and activation process (Scheme 1). The resulting carbons were evaluated for their structural and functional properties, with particular focus on optimisation of pore architecture for hydrogen storage.

## 2. Materials and methods

All metal salts were purchased from Merck and were used without any further purification. Kale (Cavolo Nero, Lincolnshire) was purchased from supermarket, and the same variety was used throughout the study for maintaining consistency.

### 2.1. Preparation of activated carbon

Chemical activation were performed using CuCl_2_·2H_2_O as an activating agent. Kale plant stems were selected as a model system for vascular biomass (10 g) and were cut into pellets of 6 mm × 2 mm (diameter × length). The stems were suspended in 50 mL of aqueous salt solution of CuCl_2_·2H_2_O with concentrations of 10, 20, 40, 80, 100, 150 and 350 mg mL^−1^ for 24 hours. A control sample was prepared by suspending identical amounts of precursor in distilled water for 24 hours. The solutions were then decanted, and the biomass were washed with distilled water and left to dry under air for 72 hours in a fume cupboard. The dried pellets were then carbonized in a tube furnace. To determine the optimum activation temperature, the pellets were activated at three different temperatures, 500 °C, 700 °C and 900 °C, as shown in Table S2. The carbon samples derived from 500 °C, 700 °C, and 900 °C showed surface area of 106 m^2^ g^−1^, 263 m^2^ g^−1^, and 438 m^2^ g^−1^, respectively. In the present work, where the focus is to study the effect of CuCl_2_ concentration on the activation behaviour, 900 °C was selected as activation temperature based on the highest surface area found in the above optimisation. After placing the dried pellets into the tube furnace, the furnace was purged with nitrogen at a constant flow rate of 128 mL min^−1^ for 30 min at room temperature, then heated to 105 °C and held for 30 min. The sample was then heated to 900 °C under nitrogen flow, where it was held for 120 minutes to produce the activated carbon. Samples are labelled as CuX-900-2, where X is the CuCl_2_·2H_2_O concentration in mg mL^−1^. The control sample (treated with distilled water only) is denoted as Control-900-2.

### 2.2. Characterisation and gas adsorption analysis

Thermogravimetric analyses (TGA) were performed using a Netzsch STA 449 F1 Jupiter. 10 mg of sample was put in an alumina crucible and heated at a rate of 10 °C min^−1^ under a nitrogen flow rate of 50 cm^3^ min^−1^. The TGA runs were divided into six segments, as described in Fig. S1 in the SI. An initial temperature ramp under nitrogen was used to remove moisture, followed by a ramping step to 900 °C to monitor the weight change during the carbonisation. After cooling under nitrogen, a switch to air and a second ramp to 900 °C was performed to assess the weight change under oxidative conditions, as well as the residual mass from inorganic content.^36^

Gas sorption measurements were performed with a Micromeritics 3Flex analyser using nitrogen (Air Liquide, 99.9999%) or hydrogen (Air Liquide, 99.9999%) at 77 K. Prior to analysis, the samples (100–200 mg) were degassed using a Micromeritics SmartVacPrep at 200 °C for 720 min under vacuum. All isotherm data were collected with filler rods inserted into the sample tubes to minimise free space, in the range *p*/*p*_0_ 0–0.995. Following analysis, free space measurements were performed using helium (Air Liquide, 99.9999%).

BET surface area were calculated in accordance to ISO 9277,^37^ with the Rouquerol criteria applied to ensure determination of surface area of microporous carbon. Pore size distributions were calculated using HS-2D-NLDFT model of carbon at 77 K fitting with slit-shaped pores using micromeritics Flex software, while the micropore volumes (*V*_μ_) of the activated carbon samples were independently determined using the Dubinin–Radushkevich (DR) method (plots shown in section 5 of the SI). Hydrogen uptakes at low pressure (<1 bar) were measured *via* volumetric H_2_ sorption at 77 K using the Micromeritics 3Flex as described above. Hydrogen (Alphagaz 2, (99.9999%) 6.0) uptake at high pressure (up to 90 bar) was measured gravimetrically at 77 K using a XEMIS-001 instrument (Hiden Isochema, United Kingdom). For room temperature adsorption measurements, the liquid nitrogen dewar was replaced with a recirculating temperature-controlled chiller set at 25 °C. This enabled the sample chamber to maintain a near ambient temperature throughout the measurement. Before analysis, leak tests were conducted, and the samples were degassed *in situ* at 200 °C for 720 min under vacuum. Skeletal volume was determined using helium (Alphagaz 2, (99.9999%) 6.0) *via* the Hiden XEMIS-001 to account for buoyancy force at high gas densities.

The high-pressure adsorption measurements give the excess uptake capacity. The total hydrogen uptake was estimated from the excess uptake using an adsorbed-phase density correction, shown in eqn (1), where *ρ*_g_ is the bulk gas density at the measurement temperature and pressure and ρ_ads_ is the assumed adsorbate density. The adsorbate density was approximated using the density of liquid hydrogen, 0.07085 g cm^−1^.1
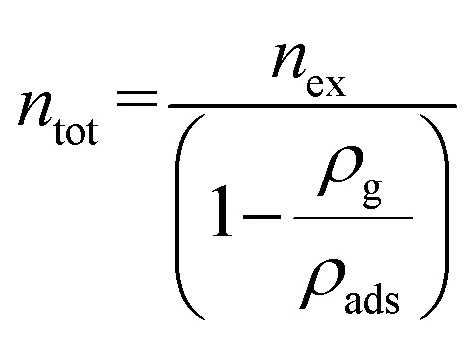
Powder X-ray diffraction (PXRD) patterns were measured using a Bruker AXS D8 Advance diffractometer, equipped with Cu Kα radiation source, at 40 kV and 40 mA at a wavelength of *λ* = 1.5406 Å, with a step size of 0.05° (2 seconds per step) and 2*θ* between 10° and 80°.

Raman spectroscopy was performed using a Renishaw Ramascope System 2000 with a 514.5 nm green laser in the range of 800 to 3000 cm^−1^. The laser excitation level was chosen at 1% to prevent thermal degradation. For each sample, spectra were collected in three randomly selected locations to minimize the effect of local heterogeneity and surface effects. The spectra were baseline corrected and normalised. The spectra from wavenumber range of 1100–1800 cm^−1^ were subjected to 4-peak fitting using a Gaussian model with MATLAB using assigned peak conventions by Sadezky *et al.*^38^ The weighted mean of the integrated peak areas (*I*_D1_/*I*_G1_) as well as the deconvoluted spectra are available in the SI.

Scanning electron microscopy (SEM) images were captured using a JEOL IT300 instrument with a working distance of 11–15 mm and an accelerating voltage of 10–15 kV. Energy dispersive X-ray data were collected using an X-Max 80 mm^2^ EDX detector and analysed with AZtec software (Oxford Instruments, UK).

Transmission electron microscopy (TEM) samples were prepared by weighing approximately 5 mg of ground activated carbon samples in 5 mL of acetone. The suspension was drop casted onto TEM copper grids that have been coated with a thin film of graphite. Samples were then imaged using a JEM-2100F instrument (JEOL, Japan) at 200 kV equipped with an Orius SC1000 camera (Gatan, US).

## 3. Results and discussion

### 3.1. Materials characterisation

**Fig. 1:**
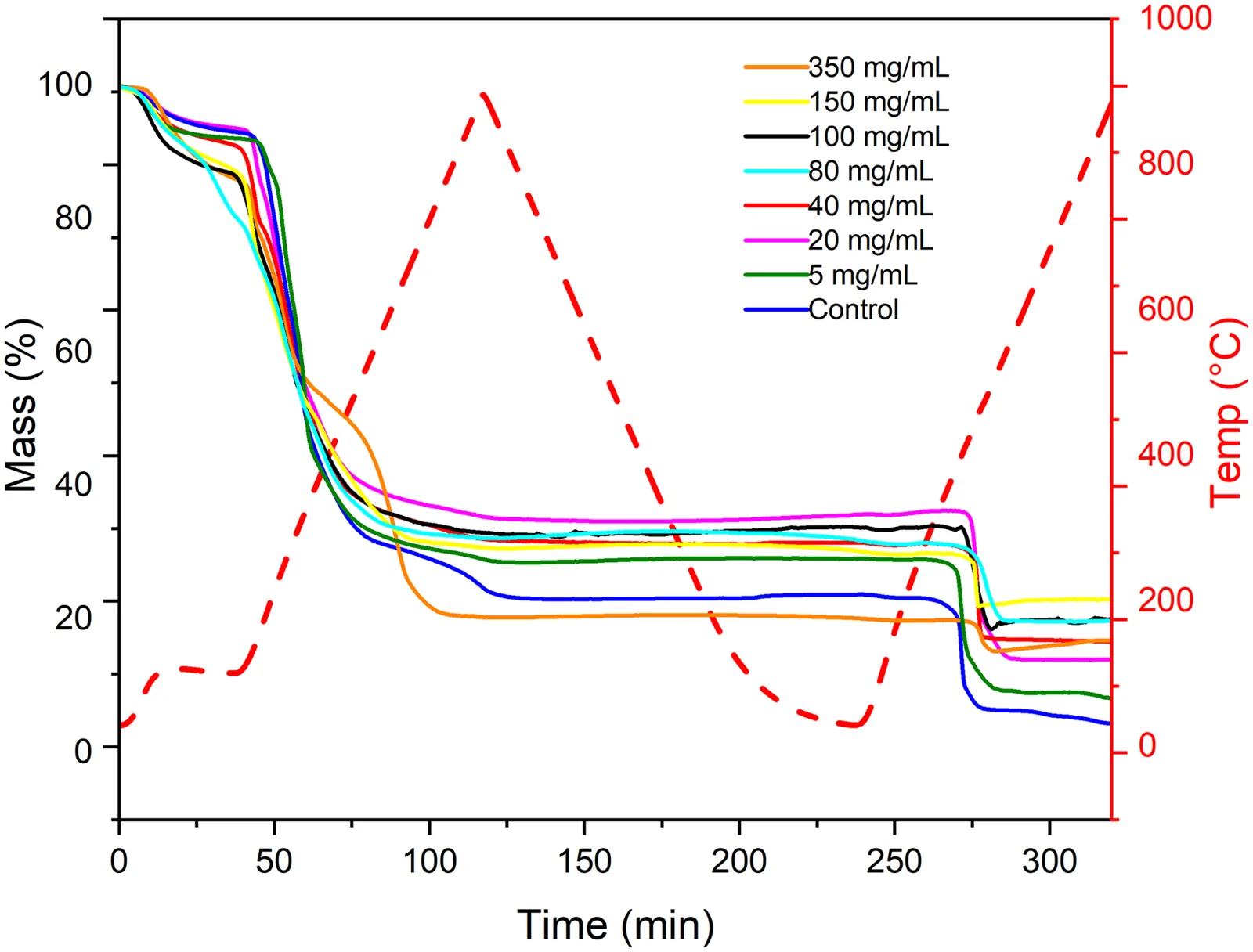
Thermogravimetric trace of kale precursors doped with different concentrations of CuCl_2_ (0–350 mg mL^−1^) with respect to time. The gas flow was switched to air at 204 min.

Thermogravimetric analysis (TGA) can give insight into the thermal stability of a material, and the residual inorganic content at the end of the carbonisation process.^39^ The biomass precursors were first heated to 900 °C in nitrogen, followed by a second step of oxidation at 900 °C in air, to determine the ash content (Fig. 1). In Fig. S2, all traces were shifted to begin at 100% after the initial isothermal hold at 100 °C (segment 2), so subsequent mass changes reflect mass change after the initial moisture loss step.

In the TGA plots shown in Fig. 1, an initial mass loss at around 100 °C corresponds to loss of water. For the control sample with a loss of ∼7.2% mass was observed around 100 °C, accounting for the moisture present in the biomass precursor. For CuCl_2_-doped precursors, the magnitude of this loss increases systematically with increasing CuCl_2_ content (shown in Table S3). This is consistent with the increasing presence of hydrated Cu^2+^ species. A dominant mass loss begins around 200 °C until approximately 500 °C, with a sharp mass loss of 58.8% for the control sample, and ranging from 56.9% to 36.0% for the samples impregnated with CuCl_2_ salt (5–350 mg mL^−1^, Table S3 in SI). This mass loss corresponds to the primary thermal decomposition of the plant biomass precursor, involving the decomposition of hemicellulose, cellulose, and lignin. Hemicellulose and cellulose typically degrade at 220–315 °C and 315–400 °C, respectively.^40^ A fixed-window TGA analysis of the kale stem biomass gave operational TGA-derived fractions that are consistent with lignocellulosic and herbaceous biomass in literature.^41^ Although a significant unassigned fraction indicates that these values should be interpreted as qualitative TGA-derived fractions rather than thermal compositional analysis (see SI section 2).

Around 450 °C, a small drop was observed primarily for the samples with more concentrated CuCl_2_, and this can be attributed to the degradation of CuCl_2_ (as seen in the TGA plot of CuCl_2_ in Fig. S3 in SI). The thermal profile of the 350 mg mL^−1^ precursor differs significantly from lower CuCl_2_ doped samples. As shown by the TGA of pure CuCl_2_ (Fig. S3 in SI), the salt undergoes decomposition between approximately 400 °C and 650 °C, which is reflected in the TGA profile for the highest doped sample, indicating that the salt loading is sufficiently high to dominate its thermal decomposition features.

Lignin decomposes at wide temperature range (160 °C and up to 900 °C),^40,42^ and the minor mass loss around 700–800 °C in Fig. 1 (more prominent in the control sample) can be attributed to the degradation of more stable lignin-derived aromatic residues. Ash content was determined by oxidation of residual carbon materials in air at 900 °C. Samples showed a sharp mass loss of 18.6% for the undoped control and loss of 20.7%, 22.2%, 14.8%, 11.7%, 14.1%, 7.4%, and 3.0% for precursors impregnated with 5, 20, 40, 80, 100, 150, and 350 mg mL^−1^, respectively.

The observed variation in mass loss behaviour with increasing CuCl_2_ are shown in Fig. S2 in SI. At low concentrations (5–40 mg mL^−1^), Cu^2+^ species promote the decomposition of lignocellulosic biomass components, enhancing the mass loss. However, the carbothermal reaction causes reduction of CuCl_2_ and leads to the formation of metallic Cu, which remains in the sample, hence contributing to an increase in the residual mass during the main carbonisation segment.

Because this oxidation step removes the remaining carbonaceous materials, a lower mass loss directly corresponds to a higher residual inorganic content. Samples with lower CuCl_2_ loading contain higher fraction of carbon. Residual content in Table S4 shows the residual mass at the end of the oxidation process. The wt% residual mass increases as the doping of the CuCl_2_ is higher, which indicates the presence of higher fraction of inorganic content, corresponding to higher presence of Cu due to the loading process.

**Fig. 2:**
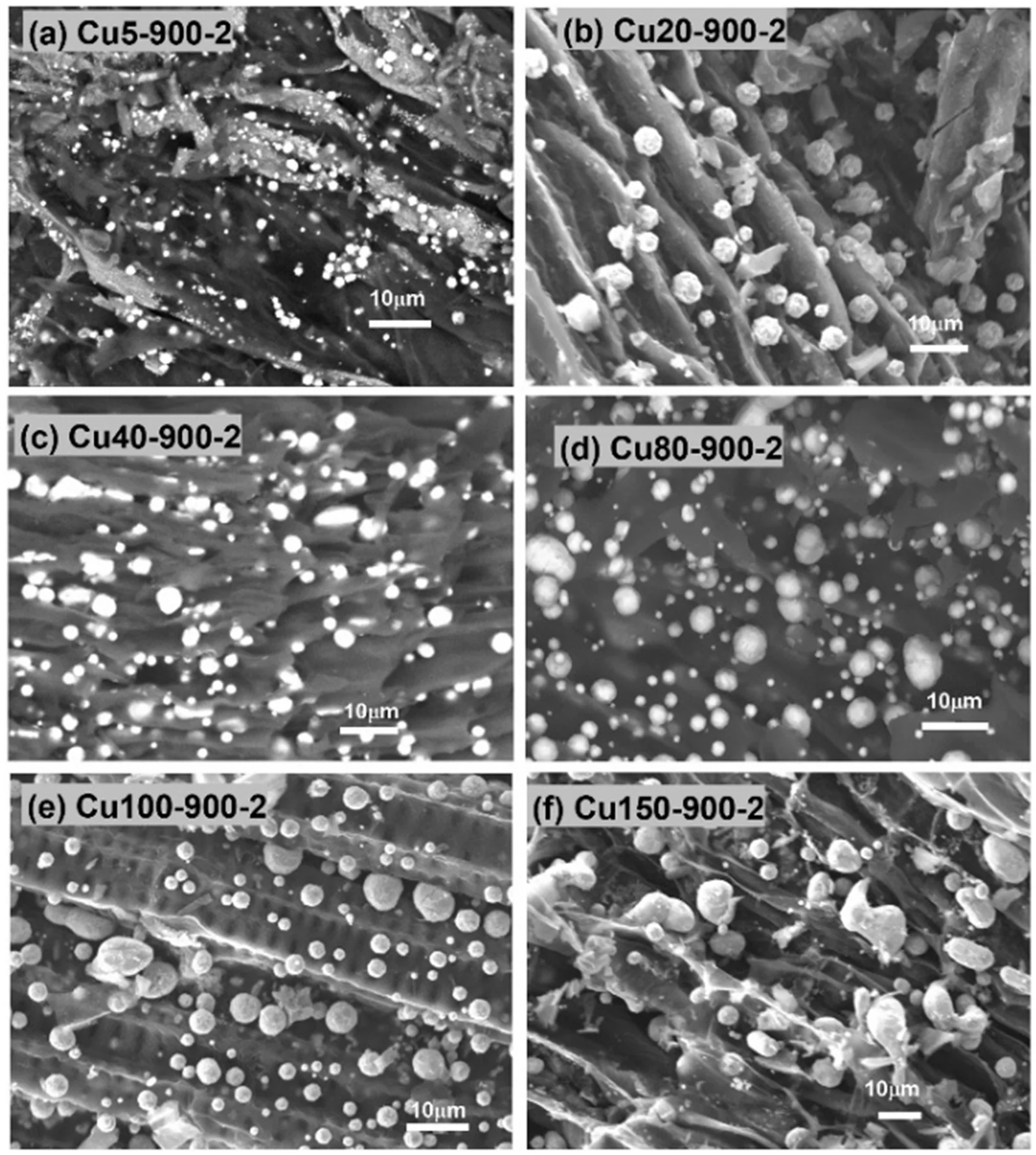
SEM images of CuCl_2_ activated carbon. (a) Cu5-900-2, (b) Cu20-900-2, (c) Cu40-900-2, (d) Cu80-900-2, (e) Cu100-900-2, (f) Cu150-900-2.

SEM images in Fig. 2(a)–(f) show the surface morphology of carbon samples activated at increasing concentrations of CuCl_2_, from 5 mg mL^−1^ (Cu5-900-2) to 150 mg mL^−1^ (Cu150-900-2). Spherical particles appear to be dispersed across the carbon matrix. A clear visual trend in the SEM images show that increasing the metal salt concentration in the precursor leads to increasing metal particle size. Fig. S5 and Table S5 in SI shows representative micrographs and Feret diameter distribution of Cu-activated samples. At low CuCl_2_ concentrations (Cu5-900-2), the particles appear finely dispersed with an average Feret diameter of 0.32 μm (average area of 0.135 μm^2^), increasing the CuCl_2_ concentration leads to a progressive particle size increase. As seen in the broader size distributions, the average Feret diameter increases from 0.96 μm to 5.69 μm and an average area from 1.13 to 38.3 μm^2^ for samples Cu20-900-2, and Cu350-900-2, respectively. This trend indicates enhanced particle sintering and growth with increasing Cu content.

**Fig. 3:**
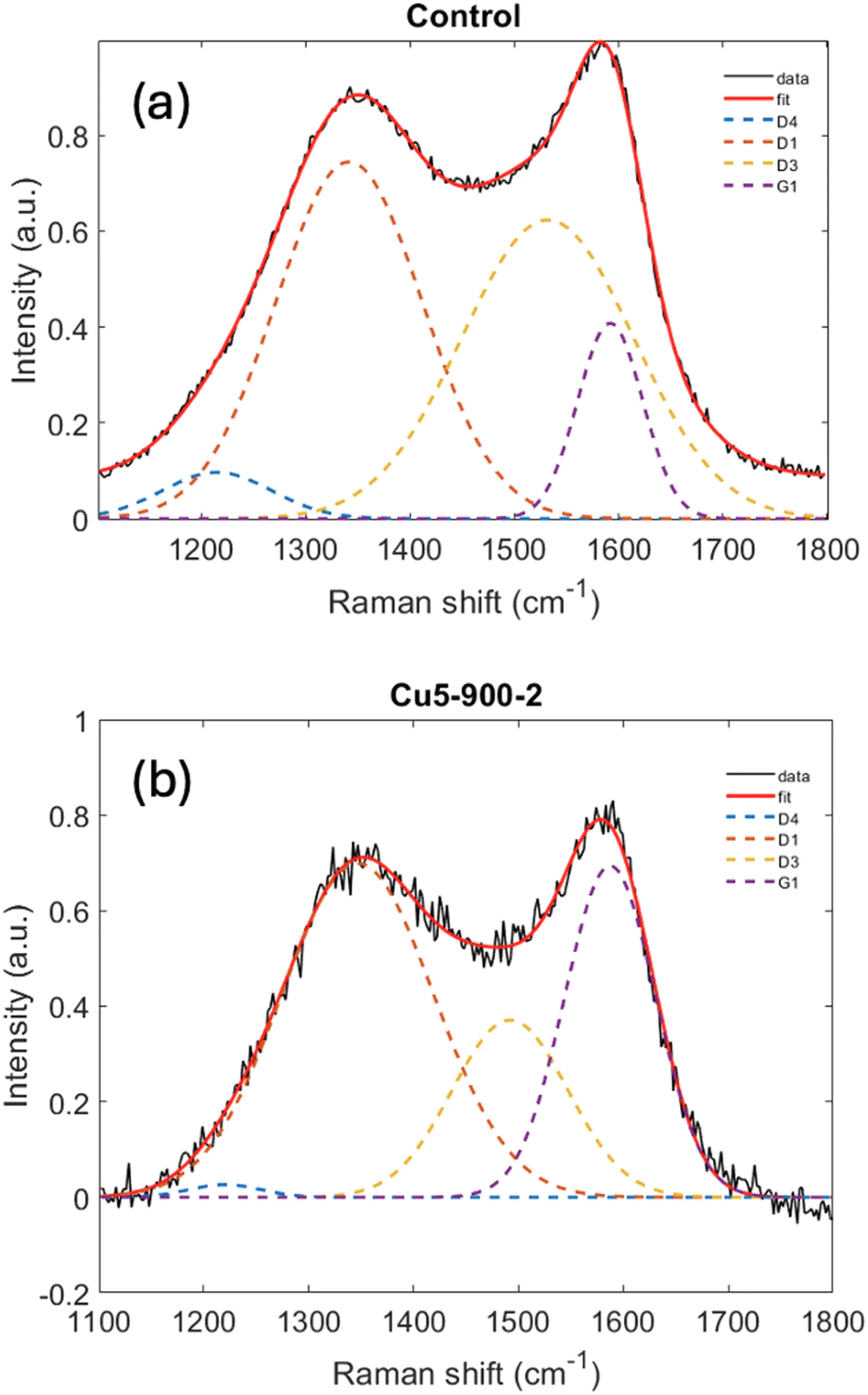
Raman spectra of (a) control sample and (b) representative CuCl_2_ activated carbon (Cu5-900-2).

PXRD results of three concentrations of CuCl_2_-activated carbon are shown in Fig. S6. XRD pattern of the control sample shows a broad diffraction feature between 2*θ* = 20° to 25°, characteristic of turbostratic carbon structure.^43^ Over this broad background, several superimposed reflections are observed, most notably at 2*θ* = 28°, 40° and 50°, corresponding to the (200), (222), and (400) crystalline planes, respectively, which are associated to the presence of potassium chloride. Additionally, a set of lower intensity superimposed peaks observed at 2*θ* = 29.6°, 31.2°, 45.0° and 58.5° correspond to the (111), (200), (220), and (222) planes of sodium chloride.^44,45^ EDX analysis also confirms the presence of Na, K and Cl as inherently present in the biomass char and abundant in leafy biomass.^46^ The CuCl_2_·2H_2_O activated samples show diffraction peaks of metallic Cu with no peaks indicating the presence of oxides, indicating that the Cu^2+^ was fully reduced to Cu during the pyrolysis process. 2*θ* peaks observed at around 43°, 51°, and 75° correspond to the (111), (200), and (220) planes respectively, indicating the *fcc* lattice structure of Cu particles and confirming the formation of crystalline Cu^0^ within the carbon lattice.^47^ EDX analysis (Fig. S7 and S8 in SI) of the activated carbons shows high intensity signals for Cu and C, which indicates the presence of Cu in activated carbon, as expected. Additionally, the samples show presence of other elements including Ca, P, S and O, which are expected to be present in plant-derived biomass materials.^48^ Raman spectroscopy was used to investigate the degree of graphitisation of porous carbon (Fig. 3). As shown in Table S2, the spectra from range 1100 to 1800 cm^−1^ were deconvoluted into 4 Gaussian peaks, assigned to D4, D1, D3, and G1 modes at 1235, 1348, 1512, and 1593 cm^−1^, respectively. Table S2 shows the fitted spectra overlayed over the Raman data, as well as the deconvoluted peaks of the three repeat measurements for all the samples, while Table S6 includes the calculated fitting parameters, FWHM and peak positions.

As shown in the Raman spectra, two characteristic G1 and D1 peaks are observed in all samples, pertaining to the graphitic and disordered nature of the carbon structure, respectively. The D1 peak corresponds to the breathing mode of the sp^2^ rings in disordered carbon around edge of the graphene layers, whereas the G1 band is a 1st order band associated to in-plane stretching of sp^2^ carbon–carbon bonds in the graphitic lattice.^49^ The additional D3 and D4 bands are associated with the amorphous carbon (indicating the presence of functional groups, and non-crystalline fragments), and the sp^2^–sp^3^ hybridised bonds in poorly ordered structures, respectively. The intensity ratio of the D1 *vs.* G1 bands (*I*_D1_/*I*_G1_) were determined from the integrated areas of the deconvoluted G1 and D1 peaks. As shown in Table 1, all samples were compared against the control sample, where the *I*_D1_/*I*_G1_ ratio is 3.31. This indicates that the kale-derived carbons are highly defective. Compared to the control, the introduction of Cu leads to an overall decrease in I_D1_/I_G1_ ratio, suggesting a Cu-induced reorganisation of the short-range sp^2^ structure towards more ordered graphitic domains. It is also noticed that the introduction of copper even at very low concentration reduced the peak intensity of the D3 bands, indicating the overall amount of amorphous carbon is reduced in the bulk phase. In addition, Table S7 shown in SI reveals a narrowing of the G1 band FWHM with increasing concentration of activating agent, indicating partial reorganisation and increased uniformity of localised sp^2^ domains.^49^ However, the D1 band FWHM remains broad and consistent across the samples, reflecting that carbon still exhibits a highly disordered, defect-rich structure. This suggests that Cu is promoting local structural rearrangements, without fully eliminating disorder during the activation process. This trend was also observed in other earlier studies.^19–21^ The graphitisation ability of Cu was also found to be dependent on factors that change with concentration, such as particle size and agglomeration.^50^

**Table 1:** *I*_D1_/*I*_G1_ ratios of activated carbon samples

Sample	*I*_D1_/*I*_G1_ ± SEID1/IG
Control-900-2	3.31 ± 0.26
Cu5-900-2	2.59 ± 0.28
Cu20-900-2	2.58 ± 0.17
Cu40-900-2	2.84 ± 0.11
Cu80-900-2	2.91 ± 0.11
Cu100-900-2	2.85 ± 0.10
Cu150-900-2	2.81 ± 0.09
Cu350-900-2	2.62 ± 0.14

**Fig. 4:**
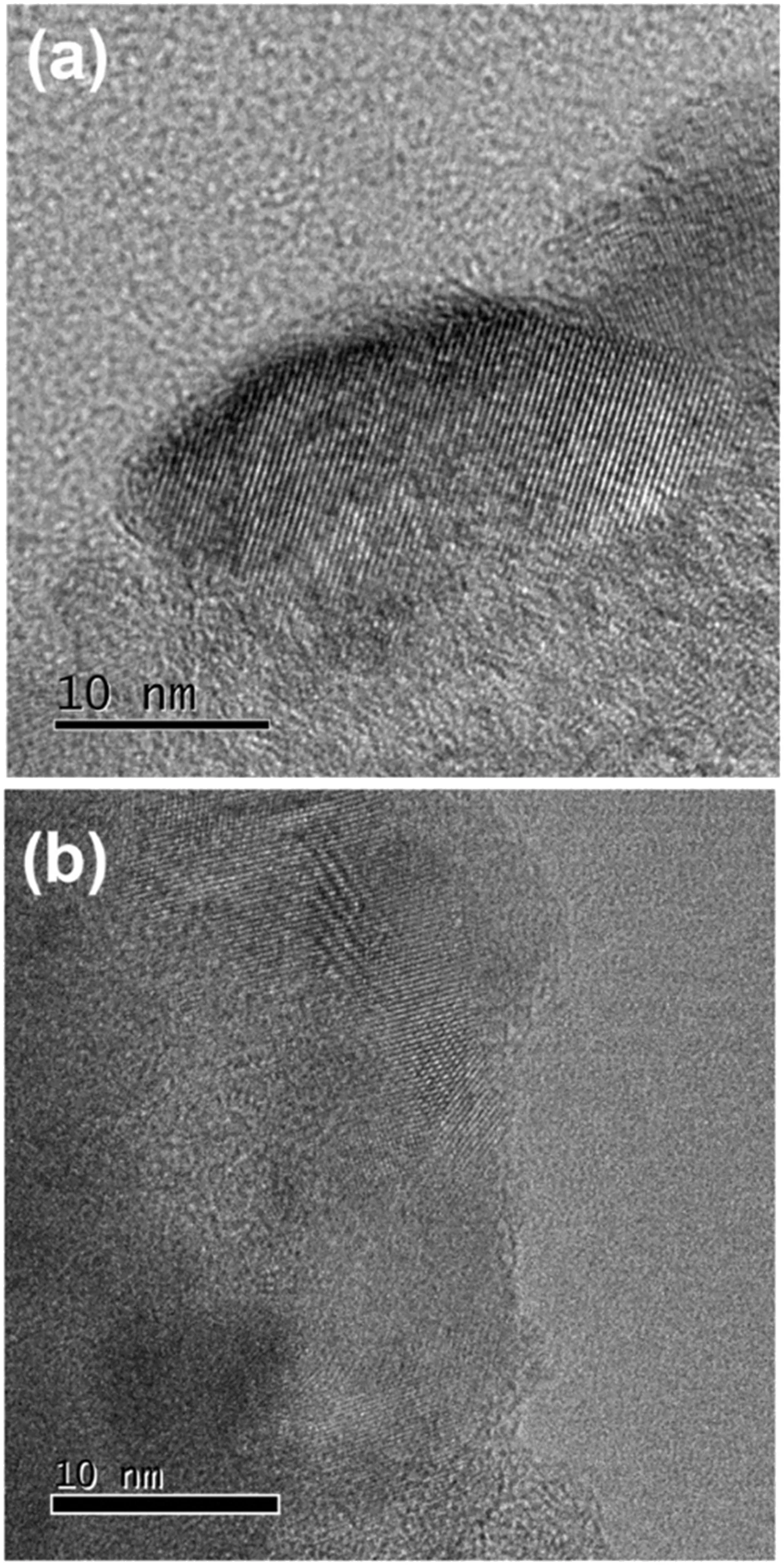
TEM images of sample (a) Cu40-900-2 and (b) Cu80-900-2, showing areas of partial graphitisation.

To complement the Raman analyses, TEM imaging was carried out to qualitatively assess the localised ordered structures of graphitic carbon. In Fig. 4 and S9, layered patterns can be observed for Cu40-900-2 and Cu80-900-2, revealing localised domains with *d*-spacing of 0.335 nm, which is consistent with the interlayer distance between graphitic (002) planes. This observation further supports the findings from Raman analyses, indicating that Cu can facilitate localised sp^2^ reorganisation. This observation is also consistent with earlier studies reporting graphene-like turbostratic domains formed *via* Cu-assisted etching at temperature ranges of 825 °C to 850 °C.^51,52^

### 3.2. Gas adsorption studies

#### 3.2.1. Surface area and porosity

**Fig. 5:**
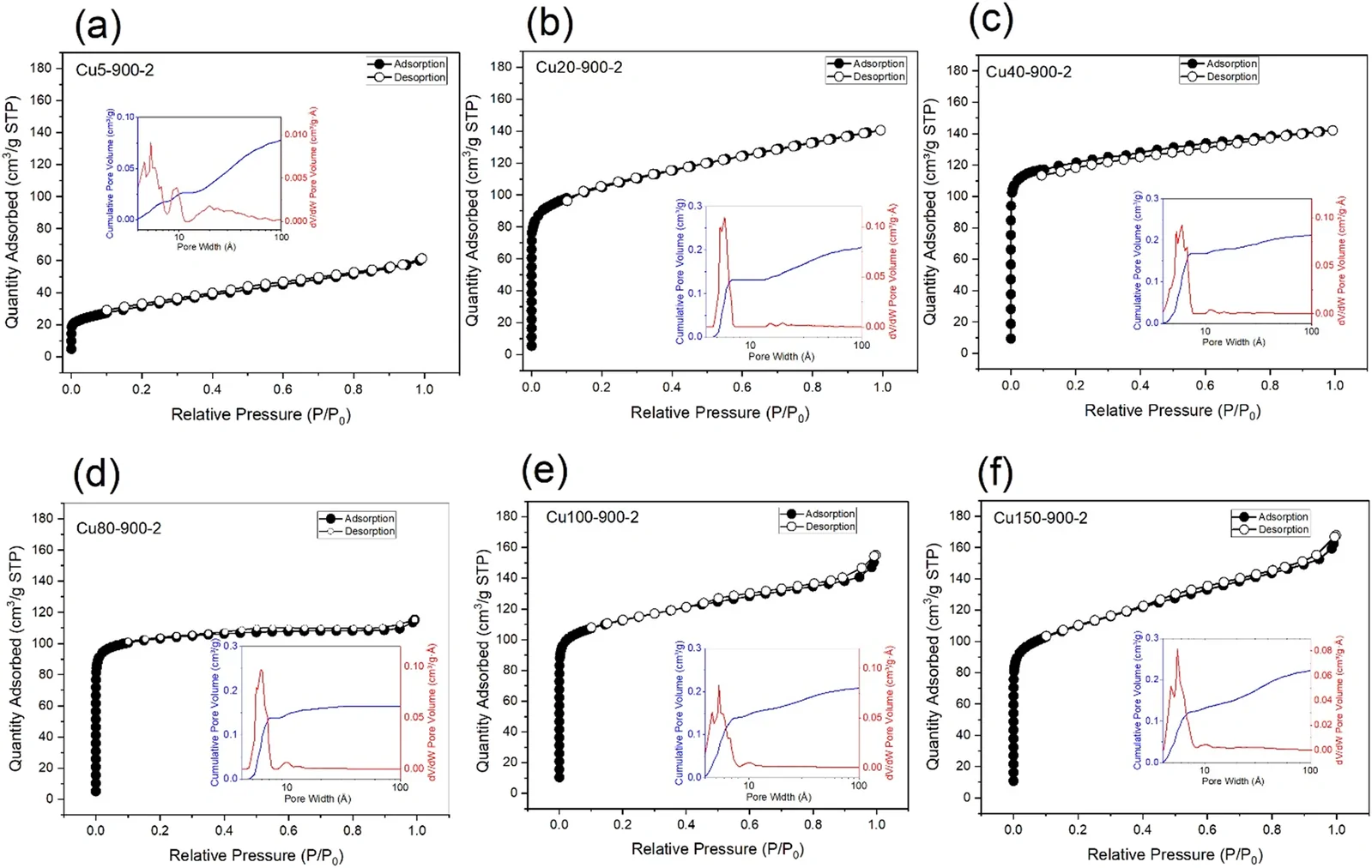
(a–f) Nitrogen sorption isotherms of CuCl_2_-activated carbon at 77 K prepared using different concentrations of CuCl_2_. Insets show the corresponding pore size distributions and the cumulative pore volume curves.

Nitrogen adsorption–desorption isotherms were measured at 77 K to investigate the porosity and surface area of the activated carbon samples, as shown in Fig. 5. As classified by IUPAC, the CuCl_2_-activated carbon shows a type I(b) isotherm, characterised by steep uptake at lower relative pressure, reaching a plateau at saturation.^53^ This is characteristic of microporous materials, as micropores tend to fill up more quickly at lower pressures than meso/macropores due to stronger adsorbent–adsorbate interactions.^54^ To calculate surface area, the Brunauer–Emmett–Teller (BET) method was employed. BET surface area of each sample was determined from linear BET plots (Fig. S10) following the Rouquerol criteria to ensure a positive correlation and the highest correlation function (*R*^2^) values. It is important to note that the applicability of the BET method to highly microporous materials is limited. As addressed in literature, nitrogen adsorption at 77 K is dominated by micropore filling rather than multilayer adsorption, which violates assumptions of the BET theory, and hence, the calculated surface area might not represent the actual physical area, but rather an apparent value that is critically dependent on the selected relative pressure range (*P*/*P*_0_ < 0.1).^53^ Pore size distributions and cumulative pore volume of samples were calculated (Fig. 5) by fitting the isotherms to the HS-2D-NLDFT model, using an asymmetric carbon slit pore shape.

**Fig. 6:**
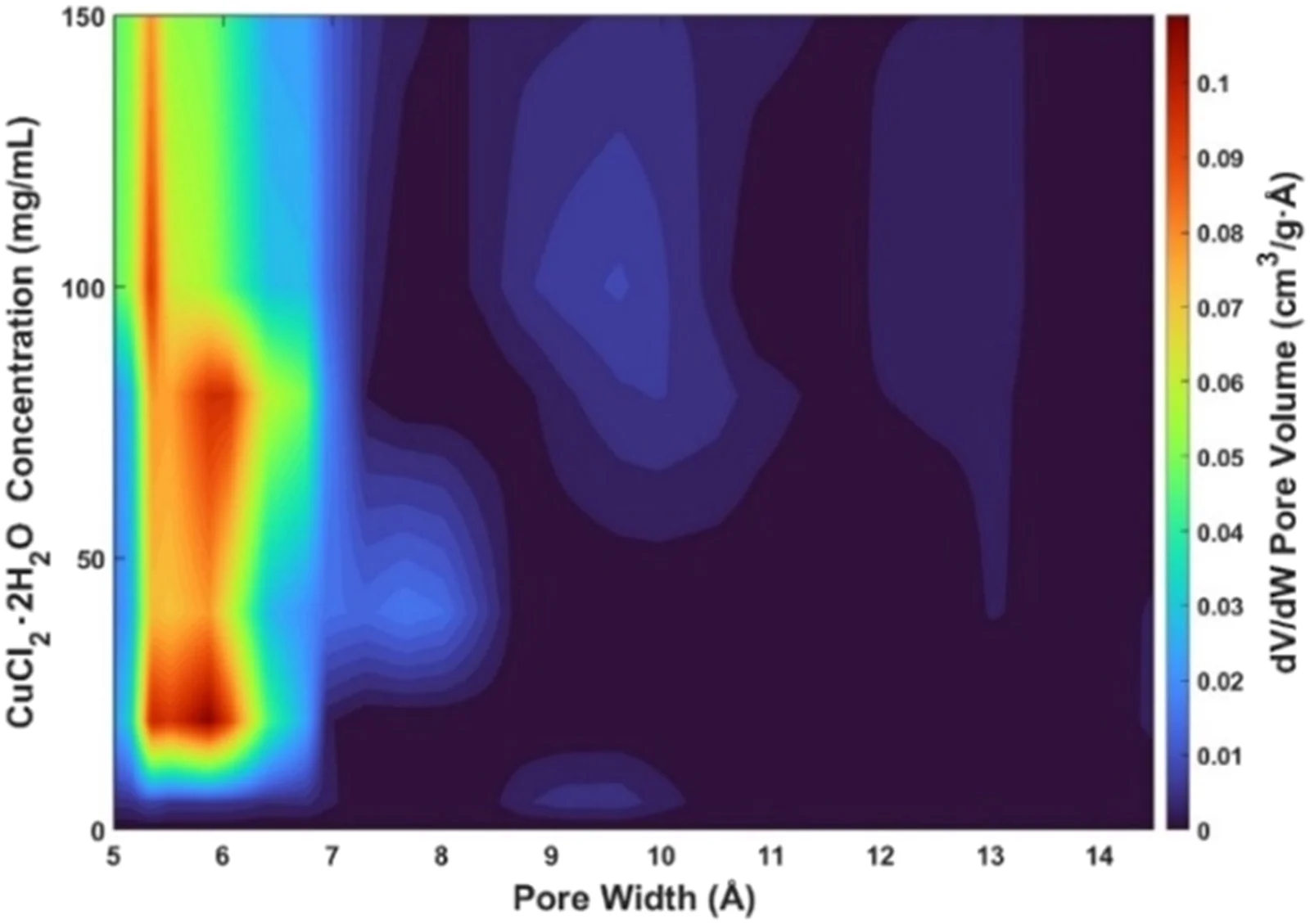
Heat map of pore size distribution (PSD) against concentration of activating agent, showcasing changes in microporosity.

The Cu5-900-2 carbon exhibits a surface area of 111 m^2^ g^−1^ with pore size distribution (PSD) centred around the microporous region, suggesting that the activation promotes pore narrowing and/or the formation of new micropores. However, this small micropore development was not found to be sufficient for any noticeable increase in surface area. At higher CuCl_2_·2H_2_O concentrations, there is a significant increase in surface area to 385.5 m^2^ g^−1^ and 437.5 m^2^ g^−1^ for Cu20-900-2 and Cu40-900-2, respectively. This indicates increased chemical activation, increasing surface area and micropore formation. A decline in surface area at much higher concentration (350 mg mL^−1^ of CuCl_2_·2H_2_O), was observed in Cu350-900-2, which can be due to the onset of overactivation (which can result in pore-widening) or agglomeration of copper particles. Additionally, the incorporated particles within the carbon framework might aid in contributing to surface area through textural enhancement of the surface.^55^ The PSD of control sample in Fig. S11 shows negligible microporosity, with no observable pores below 10 Å. Instead, it is dominated by larger mesopores, primarily ranging between 20 Å and 100 Å. In contrast, CuCl_2_ activated samples show strong presence of micropores in a range between 5 Å and 7 Å. Although according to IUPAC, micropores are defined as pores with widths less than 20 Å, pores below 10 Å are often referred to as ultramicropores which are essential for hydrogen adsorption, as the confined pore walls in this region result in higher adsorbent-adsorbate interactions due to overlapping potential fields.^56,57^ Relative to the control, increasing the CuCl_2_·2H_2_O concentration leads to a pronounced increase in porosity around 6 Å pore diameter, reaching a maximum for sample Cu20-900-2. This trend is evident in both the pore size distribution plots and the contour map (Fig. 5 and 6), indicating optimal ultramicroporosity obtained with moderate concentrations of activating agent (Table 2).^58^

**Table 2:** Table showing BET surface area, micropore volume and average micropore width of activated carbons prepared with varying concentrations of CuCl_2_ loadings and carbonised at 900 °C for 2 hours

Sample	Mean BET surface area (m^2^ g^−1^)	Micropore volume^a^ (cm^3^ g^−1^)	Average micropore width^b^ (Å)
Cu5-900-2	111.5 ± 0.6	0.05	8.64
Cu20-900-2	385.5 ± 0.9	0.16	4.02
Cu40-900-2	437.5 ± 0.5	0.18	4.17
Cu80-900-2	404.4 ± 0.9	0.16	3.28
Cu100-900-2	427.9 ± 0.7	0.17	5.02
Cu150-900-2	404.9 ± 0.5	0.17	4.02
Cu350s00-2	206.3 ± 0.9	0.08	6.07

a Calculated from fitting Dubinin–Radushkevich (DR) fitting.

b Volume weighted average pore size below 20 Å.

Further increase in CuCl_2_·2H_2_O concentration results in reduction and broadening of the ultramicroporous peak at 6 Å, which can be attributed to several concurrent effects. Firstly, overactivation may occur, whereby smaller pores start to merge into larger mesopores, leading to a decrease in overall micropore volume. Secondly, the presence of Cu derived species in larger amounts can reduce the accessible pore volume by partially occupying pore spaces and entrances.^59^ Thirdly, the presence of excess activating agent can weaken the carbon framework, causing partial collapse of the micropores, and the loss of well-defined microporosity.^8,60^ The trend in porosity was further studied using Dubinin–Radushkevich (DR) model (Fig. S12). Unlike surface-based adsorption isotherms like BET and Langmuir, the DR isotherm model describes the adsorption behaviour of micropores by pore filling, and is governed mainly by adsorption potential rather than surface coverage.^61^ In this model, the amount of adsorbate relates to the adsorption potential, allowing for an estimate of micropore volume (*V*_μ_) and characteristic adsorption energy (*ε*).^62^ All samples exhibit good linearity, with high correlation coefficients (*R*^2^ ≈ 0.92–0.99), indicating that micropore filling is the dominant adsorption mechanism in the low relative pressure region.^63^ The control sample exhibits low micropore volume (*V*_μ_ ≈ 0.053 cm^3^ g^−1^) as well as characteristic adsorption energy, (*ε* ≈ 3.7 kJ mol^−1^), which further highlights the limited microporous nature shown in the pore size distribution. In contrast, CuCl_2_·2H_2_O activated samples display significantly higher micropore volumes and characteristic adsorption energies, reflecting the enhanced development of micropores, reaching a maximum for Cu20-900-2 and Cu40-900-2 (*V*_μ_ ≈ 0.16–0.18 cm^3^ g^−1^), in agreement with the PSD analyses. The corresponding increase in ε to around 6 to 8 kJ mol^−1^ indicates stronger adsorbate-adsorbent interactions associated with narrower micropores. At higher concentrations, notably Cu350-900-2, a reduction in micropore volume and adsorption energy is observed, consistent with over-activation effects such as pore widening, blockage, or partial collapse, as inferred from the broadening of ultramicropore peaks in the pore size distributions.^64,65^

In summary, the BET surface area increase from 100 m^2^ g^−1^ to a maximum of 440 m^2^ g^−1^ for Cu5-900-2, and Cu40-900-2, respectively, while the micropore volume follows a similar trend from 0.05 to 0.18 cm^3^g^−1^. At higher CuCl_2_ loading, the measured microporosity plateaus, showing that there is an optimal activation effect. This behaviour is consistent with excessive Cu loading resulting in possible widening and loss of ultramicropore definition, partial occupation and pore blockages by the Cu species.

Combined structural and spectroscopic analyses demonstrate that CuCl_2_ plays a critical role in modifying the structure and porosity of the carbon. This indicates that CuCl_2_ can participate in a redox-assisted activation, which is consistent with literature reports on transition metal mediated carbothermal reactions.^66^ PXRD confirms the formation of metallic Cu after pyrolysis, indicating the reduction of Cu^2+^ during the carbonisation process. eqn (2) shows a representative proposed pathway from literature.^19^ The removal of carbon atoms in this process generates micropores and leads to increases in surface area. PXRD analysis confirms the presence of metallic Cu suggesting that Cu^2+^ is reduced during the carbonisation process.

2 3CuCl_2_ + C + 2H_2_O → Cu + 2CuCl + CO_2_ + 4HCl

Based on the above mechanism, in addition to its role as an activation agent, Cu may also act as a catalyst, promoting the structural reorganisation of the carbon matrix *via* catalytic graphitisation mechanisms.^67^ Consequently, CuCl_2_ is shown to have a concentration-dependant dual role during carbonisation, acting as a chemical etchant that generates porosity and a catalysing agent that promotes structural reorganisation of the carbon matrix.

#### 3.2.2. Hydrogen sorption

**Fig. 7:**
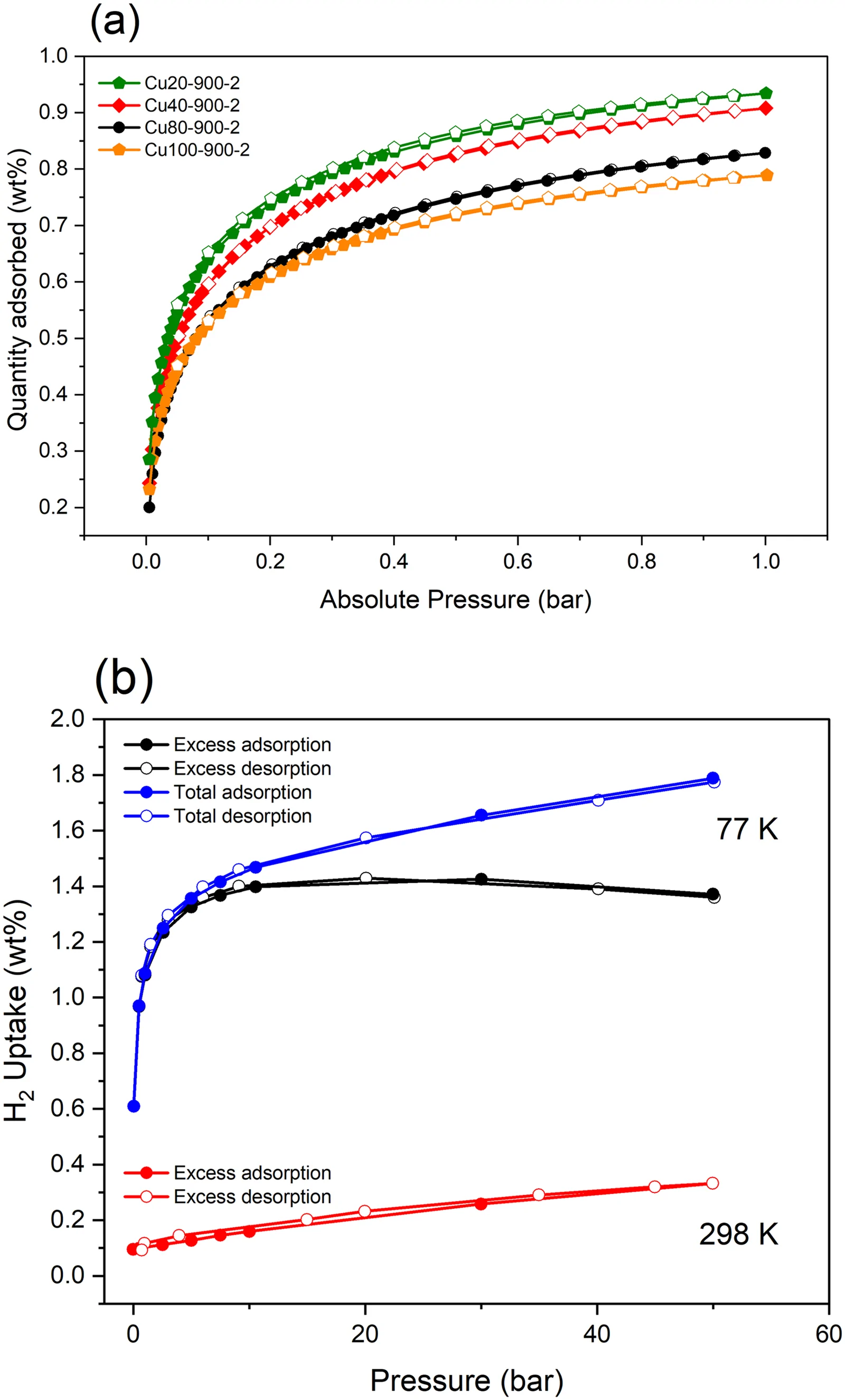
(a) Hydrogen adsorption isotherms at 77 K for Cu20-900-2, Cu40-900-2, Cu80-900-2, and Cu100-900-2 samples. (b) Adsorption–desorption isotherms of the Cu20-900-2 sample up to 55 bar at 77 K and 298 K, respectively. Showing the total and excess hydrogen adsorption isotherms.

H_2_ sorption isotherms at 77 K for the four samples with high BET surface areas are shown in Fig. 7(a). No hysteresis is evident, indicating that the hydrogen is adsorbed reversibly *via* physisorption. The low-pressure isotherms exhibit Type I shape, indicating monolayer formation, primarily due to micropore filling. Table 3 shows the hydrogen uptake at 77 K and 1 bar for the four carbon samples, indicating a modest volumetric uptake of around 0.98 wt%, 0.91 wt%, 0.83 wt%, and 0.82 wt%, for Cu20-900-2, Cu40-900-2, Cu80-900-2, and Cu100-900-2, respectively. To put these values into context, Table S8 in SI shows a comparison of the hydrogen storage performance of the activated carbon represented in this work with others reported in literature.^68^

**Table 3:** Hydrogen storage capacities of Cu40-900-2, Cu80-900-2, and Cu100-900-2 measured at 1 bar and 77 K

Sample	Hydrogen uptake at 1 bar and 77 K (wt%)
Cu20-900-2	0.98
Cu40-900-2	0.91
Cu80-900-2	0.83
Cu100-900-2	0.82

Activated carbon prepared using strong chemical activators like KOH typically exhibit higher BET surface area usually exceeding 1500 m^2^ g^−1^ and micropore volume above 0.6 cm^3^ g^−1^, resulting in H_2_ uptakes of around 2–4 wt% at 1 bar and 77 K. In contrast, this work presents CuCl_2_ activated carbon that exhibit lower surface area while maintaining moderate micropore volumes of around 0.16 to 0.18 cm^3^ g^−1^, yielding a hydrogen uptake capacity of around 0.98 wt% for the highest surface area sample (Cu20-900-2). Although the relative uptakes are lower than reported literature values, these results are notable given the one-step activation strategy and the choice of CuCl_2_ as a milder activating agent. While the CuCl_2_ activated carbon in this work show competitive uptake when normalised by BET surface area, a practically more relevant metric for hydrogen storage would be the absolute gravimetric capacity. In this light, the materials in this work remain below the benchmark of KOH-activated carbon, which usually achieve much higher H_2_ uptake under comparable cryogenic conditions. In the present study, the best CuCl_2_-activated samples exhibit BET surface area ranging from 400 to 440 m^2^ g^−1^ and micropore volumes of around 0.16 to 0.18 cm^3^ g^−1^. Hence, the results of this study demonstrate the feasibility of one-step CuCl_2_ activation strategy for generating ultramicroporous biomass-derived carbon, rather than a synthesis route that outperforms other activation strategies.

When hydrogen storage capacity at 77 K and 1 bar is normalised against BET surface area (Table S8 in SI), the materials reported in this work exhibit competitive efficiency (1.8–2.5 wt% per 1000 m^2^ g^−1^), exceeding several high-performance activated carbon reported in literature (0.9–1.7 wt% per 1000 m^2^ g^−1^).^69–71^

This shows the effective utilisation of surface area resulting from the predominance of micropores and ultramicropores (<7 Å). As it has been observed in earlier studies, there is a stronger correlation between micropore volume and hydrogen storage capacity than surface area.^72,73^ Additionally, earlier studies have shown that the presence of Cu can directly enhance the hydrogen uptake through modest enhancement of metal-hydrogen interactions and polarisation effects at cryogenic temperatures, while still maintaining fully reversible sorption mechanism.^74,75^ To assess the storage capacity at higher pressure, supercritical high-pressure gravimetric H_2_ gas sorption studies were conducted up to 50 bar at 77 K and 298 K on Cu20-900-2. The excess H_2_ adsorption (*n*_ex_) is presented in Fig. 7(b), reaching a plateau uptake of 1.33 wt% at 20 bar, where the excess uptake is defined as the amount of hydrogen adsorbed on the internal surface of the porous materials above the amount that would be present as free gas occupying the same pore volume at the same temperature and pressure.^7,76^ This indicates the contribution of small pore filing at pressures up to 20 bar, and the reduced contribution of the surface adsorption at higher pressures. When considering the total hydrogen storage capacity (*n*_tot_), the uptake increases continuously with pressure, reaching a maximum of 1.78 wt% at 50 bar. This difference between the total and excess capacity reflects the reduced contribution of surface adsorption, overweighed by the effect of hydrogen compression inside pore spaces.

Gravimetric uptake at ambient temperature for Cu20-900-2 was measured at 298 K (Fig. 7b). The uptake shows a linear relationship with increased pressure reaching an excess uptake of 0.33 wt% at 50 bar. This reduced uptake is typical of ambient isotherm measurements. At such higher temperatures, the thermal energy of the gas molecules dominates over the adsorbate-adsorbent interactions, and since adsorption is an exothermic process, this leads to weaker isosteric enthalpy, a weaker hydrogen uptake, and a behaviour that can be approximated to an ideal gas.^77,78^

Given the little-to-no hysteresis between the adsorption and desorption isotherms at low temperatures, there is no significant evidence of any strong chemisorption interactions between hydrogen and the sample. The uptake within the low-pressure regime can be largely attributed physisorption of H_2_ to the surface of the adsorbate. Although the total hydrogen uptake of 1.78 wt% at 50 bar and 77 K for Cu20-900-2 sample is smaller than the values reported for other activated carbons, it is important to consider that this activation strategy results into higher H_2_ uptake capacity when compared to activated carbons with similar surface areas. There are several examples of benchmark carbon materials with storage capacity >5 wt%, and surface areas exceeding 3000 m^2^ g^−1^.^79,80^ In contrast, our results indicate that despite the relatively moderate surface area of Cu20-900-2, the material shows highly ultramicropore-dominated structure which is essential for hydrogen storage at lower pressures, as can be evidenced from the pore size distribution. This work therefore highlights the trade-off between hydrogen storage capacity and surface area with a higher emphasis on prioritising ultramicropore formation which was achieved using metal ion impregnation of biomass precursors.

## Conclusion

This study presents an approach to highly ultramicroporous carbon synthesis, using kale stems as a biomass-derived precursor and CuCl_2_ as a mild activating agent. By varying the CuCl_2_ concentration, the extent of pore development could be systematically tuned, with optimal activation yielding a predominantly ultramicroporous structure which is efficient for hydrogen adsorption at cryogenic conditions: the optimised sample exhibits a BET surface area up to 437 m^2^ g^−1^ and a total hydrogen uptake of 1.78 wt% at 50 bar and 77 K. Thermogravimetric analyses and BET surface area analyses show that increasing CuCl_2_ concentration enhances microporosity up to an optimal threshold, beyond which possible structural collapse results into reduced microporosity. Raman spectroscopy, PXRD and TEM analyses further show the dual role of CuCl_2_ as an activating agent as well as a graphitisation catalyst, with I_D1/_I_G1_ values showing increased graphitisation with increasing CuCl_2_ concentration up to an optimal level. By demonstrating the efficacy of CuCl_2_ in engineering ultramicroporosity, this work offers a sustainable and tuneable strategy for refining the material properties of biomass-derived carbons for advanced hydrogen storage applications.

## Author contributions

L. A. M. M.: experimental design, material synthesis, and characterization, carried out characterization, prepared figures and wrote the initial manuscript draft. L. S. & C. D. B.: material characterization. M. K.: designed the experiments, performed the material synthesis, and carried out characterization. S. R.: editing and revision supervision. V. P. T.: editing and supervision. D. J. F.: editing and supervision. J. L. R.: editing and revision supervision. S. N.: conceptualisation of the project, editing, revision and supervision.

## Conflicts of interest

There are no conflicts to declare.

## Supplementary Material

LF-003-D6LF00048G-s001

## Data Availability

Data are available at the University of Bristol data repository, data.bris, at https://doi.org/10.5523/bris.1i0mx9mpjgfdq22tes4dijws15. Supplementary information (SI) with TGA, EDX, particle size analysis, Raman, and gas adsorption data are available. See DOI: https://doi.org/10.1039/d6lf00048g.
