# Supplementary material for: Copper salt impregnated biomass-derived microporous carbon for hydrogen storage
Source: RSC Appl Interfaces. 2026 May 15;3(4):1208–19. doi: 10.1039/d6lf00048g (PMC13592565; doi:10.1039/d6lf00048g)
Supplement: Supplementary file 1 [file LF-003-D6LF00048G-s001.pdf]

Supplementary Information (SI) for RSC Applied Interfaces.
This journal is © The Royal Society of Chemistry 2026

Copper Salt Impregnated Biomass-Derived Microporous Carbon for Hydrogen Storage

Lila A. M. Mahmoud,[a] Charles D. Brewster,[b] Lui Skytree,[b] Misbah Khan,[c] Sebastien Rochat,[a,d,g] Valeska P. Ting,[d,f] David J. Fermin,[a] Jemma L. Rowlandson,[d,g*], and Sanjit Nayak[b,d*]

[a.] *School of Chemistry, Cantocks Close, University of Bristol, Bristol BS8 1TS, UK*
[b.] *School of Civil, Aerospace and Design Engineering, University of Bristol, Bristol, BS8 1TR, UK. E-mail: s.nayak@bristol.ac.uk*
[c.] *School of Chemistry and Biosciences, University of Bradford, UK, BD7 1DP*
[d.] *Bristol Composites Institute, University of Bristol, Bristol, BS8 1TR, UK*
[e.] *School of Electrical, Electronic and Mechanical Engineering, University of Bristol, Bristol, BS8 1TR, UK. E-mail: J.Rowlandson@bristol.ac.uk*
[f.] *Research School of Chemistry, Australian National University, Canberra ACT 2601, Australia.*
[g.] *School of Engineering Mathematics and Technology, University of Bristol, Bristol, Bs8 1TW, UK.*

Contents:

1. Table comparing types of carbon materials

2. Thermogravimetric analysis

3. Particle size determination

4. Powder X-ray diffraction data

5. Elemental analysis and TEM imaging

6. Raman spectroscopy

7. BET analysis

1. Table comparing types of carbon materials

Table S1. *Comparison of representative carbon materials for hydrogen storage, including graphene-based, template-derived and activated carbon, showing their surface area, synthesis method, precursor, and hydrogen storage capacity.*

Carbon type	Precursor	Preparation	Surface area (m^2 g^{-1})	Hydrogen storage capacity (wt%)	Reference
**Graphene-based**	Graphite (via graphene oxide)	Thermal exfoliation of GO in H_2 atmosphere (200–500 °C)	248 (optimal at 300 °C)	3.12 wt % (77 K , 30 bar)	(Singh & De, *Mater. Chem. Phys.*, 2020)[1]
**Nitrogen- and metal-doped mesoporous templated carbon**	Acetonitrile	CVD using surfactant-modified alumina (AlS) hard template; Ni or Pd impregnation; HF template removal	1508 (N-doped); 817 (2 wt % Pd-N codoped)	5.0 wt % (−196 °C, 25 bar, 2 wt % Pd-N codoped); 3.9 wt % (N-doped)	(Singh & De, *Int. J. Energy Res.*, 2019)[2]
**Zeolite-templated carbon**	Furfuryl alcohol	Liquid phase impregnation (LPI), polymerisation and carbonisation within zeolite template followed by HF etching	3590	5.5 wt% (77 K, 20 bar)	(N. P. Stadie et al. Langmuir, 2012) [3]
**Carbide-derived carbon**	SiC, TiC	High-temperature chlorination (Cl_2 etching) of metal carbides; optional steam/CO_2 activation	1200–2200	Up to 6.7 wt % (298 K, >120 MPa, absolute uptake); ~1.2 wt % excess	(Naheed *et al.*, IJHE, 2021)[4]
**Carbide-derived**	Molybdenum carbide (Mo_2C)	High-temperature chlorination of molybdenum carbide followed by H_2 annealing	1100–2500	4.3 wt % (196 K, 35 bar)	(Kim *et al.*, *Microporous Mesoporous Mater.*, 2009)[5]
**Activated carbon**	Amorphous cellulose	Activated at 900°C and hold time for 1 hour	1499	2.36 wt% (77 k , 1 bar)	*(Conte et al., International Journal of hydrogen energy, 2024)*[6]
**Activated carbon**	Cellulose	KOH chemical activation	Around 3000	3 wt%	*(Sevilla & Fuertes, Carbon, 2009)*[7]
**Activated carbon**	Lignin	Activated at 600 °C for 2 hours using KOH chemical activation	2049	2.42 wt% (77 k , 1 bar)	*(Beak et al., Journal of*

					Environmental Engineering, 2025)[8]
**Activated carbon**	Corncob	Activated at 124 °C suing KOH chemical activation	3708	3.21 wt% (77 k ,1 bar)	*(Liu et al., Microporous and mesoporous materials, 2014)*[9]

2. Thermogravimetric analysis

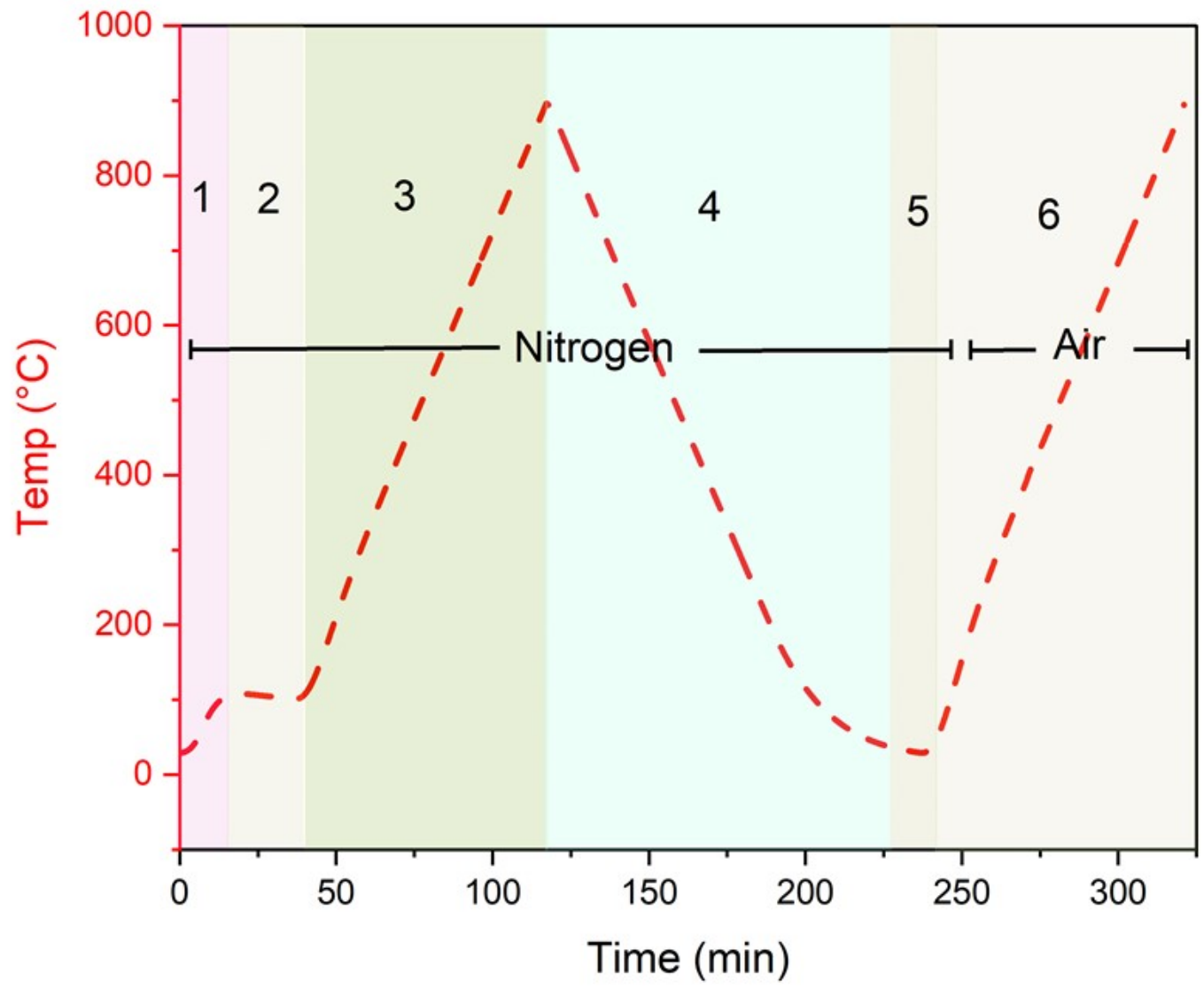

Figure S1. *Temperature program for TGA measurements showing segments 1-6, where segment (1): initial heating under nitrogen, (2) isothermal hold at 100 °C for 30 minutes, (3) ramp 900 °C under nitrogen, (4) cooling to 30 °C under nitrogen, (5) transition to air and an isothermal hold of 30 minutes, and (6) final heating to 900 °C under air*

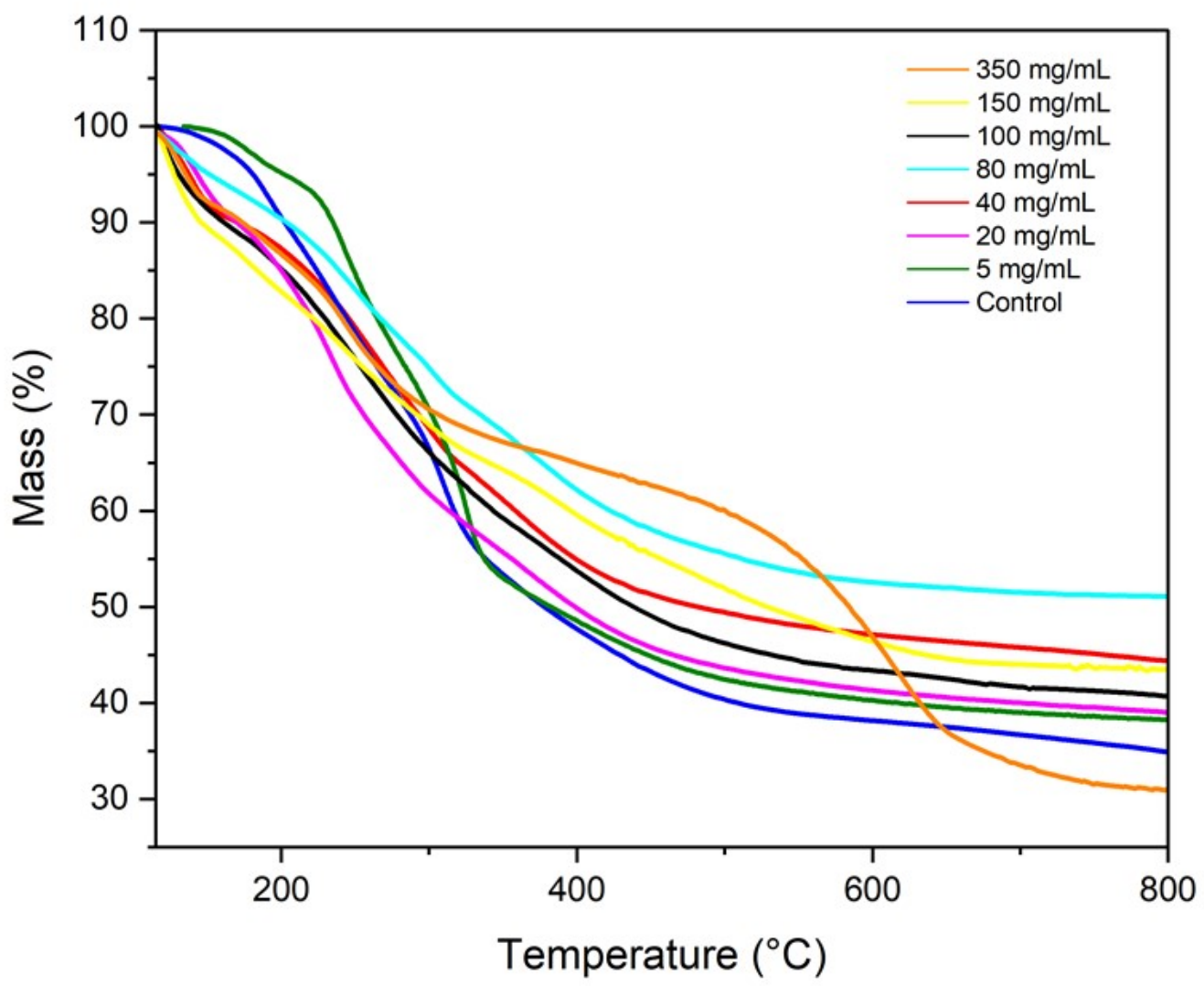

.

Figure S2. *Thermogravimetric trace of kale precursors doped with different concentrations of* $CuCl_2$ *(0 – 350 mg/mL)*

Table S2. *BET surface area of $CuCl_2$ activated biomass at 40 mg/mL at multiple temperatures.*

**Activation temperature (°C)**	**BET surface area (m^2g^{-1})**
500	106
700	263
900	438

Table S3. Mass loss (%) of kale precursors doped with 0–350 mg mL^{-1} $CuCl_2{\cdot}2H_2O$ in selected temperature regions under N_2 and air.

	Concentration of $CuCl_2{\cdot}2H_2O$/ mg/mL							
Temperature range/°C	0	5	20	40	80	100	150	350
30 - 100 under N_2	7.15	8.00	6.70	10.90	21.67	16.42	15.39	14.84
200- 500 under N_2	58.67	56.94	51.86	50.35	40.66	44.46	43.96	36.03
700-800 under N_2	3.72	2.40	2.59	3.19	1.75	2.43	3.66	18.48
300-900 under Air	18.64	20.80	22.25	14.78	11.65	14.06	7.39	3.01

Table S4. *Residual content after segment 6 (thermolysis under air)*

Concentration of $CuCl_2{\cdot}2H_2O$/ mg/mL	Residual content, wt%
0	4.39
5	8.14
20	14.00
40	16.68
80	19.79
100	19.96
150	23.06
350	16.85

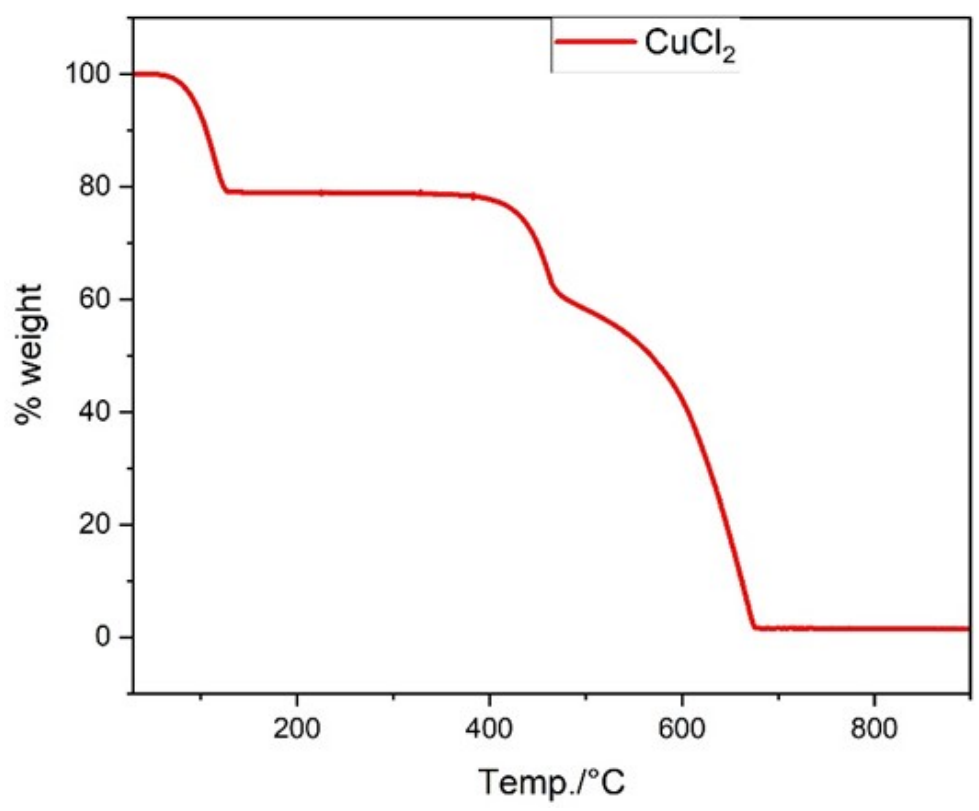

Figure S3. *TGA for $CuCl_2$ from 30 °C to 900 °C under nitrogen*

2.1 Method for estimation of lignocellulosic-component composition of kale plant biomass

Thermogravimetric analysis (TGA) was used to evaluate the thermal decomposition behaviour of biomass under inert and oxidative atmosphere. Under nitrogen, the biomass exhibits and overlapping decomposition behaviour of hemicellulose, cellulose and lignin, after the initial moisture removal step. In general, hemicellulose decomposes first, cellulose degrades over a narrower temperature interval, and lignin decomposes over a much broader range that can extend to much higher temperatures. For the analysis, replicate biomass TGA were analysed under N_2 to obtain TGA thermograms, while separate TGA runs in air were used to estimate the ash from the residual mass after oxidising at 900 °C. Because plant biomass is chemically complex, and may also contain components like pectin, minerals and other extractives in addition to cellulose, lignin and hemicellulose, the TGA-derived fractions were interpreted as component-associated fractions rather than treated as direct wet-chemical method values.

Before the analyses, the raw TGA data were normalised using initial and final masses of the TGA run. Moisture loss was excluded by applying a temperature cut-off at 150 °C and all subsequent calculations were performed on the post-moisture portion of the curve.

2.2 Window-based fraction method

Nitrogen TGA curves were analysed using a fixed-temperature window approach to obtain component-associated fractions. After exclusion of moisture loss below 150 °C from the calculations, the normalised TGA curve was divided into three intervals corresponding to hemicellulose-dominated (180–260 °C), cellulose-dominated (260–360 °C), and lignin-dominated/high-temperature (360–700 °C) decomposition. These windows were selected based on the established thermal behaviour of lignocellulosic biomass, in which hemicellulose generally decomposes at lower temperature, cellulose exhibits a sharper intermediate-temperature event, and lignin degrades over a broad higher-temperature range with substantial overlap. The assigned fraction for each interval was calculated from the mass loss across the corresponding window, and the residual unassigned fraction was retained to account for overlap and non-lignocellulosic decomposition. Because biomass also contains components such as pectin, proteins, pigments, extractives, and minerals, these results were interpreted as TGA-derived fractions rather than direct wet-chemical composition value.

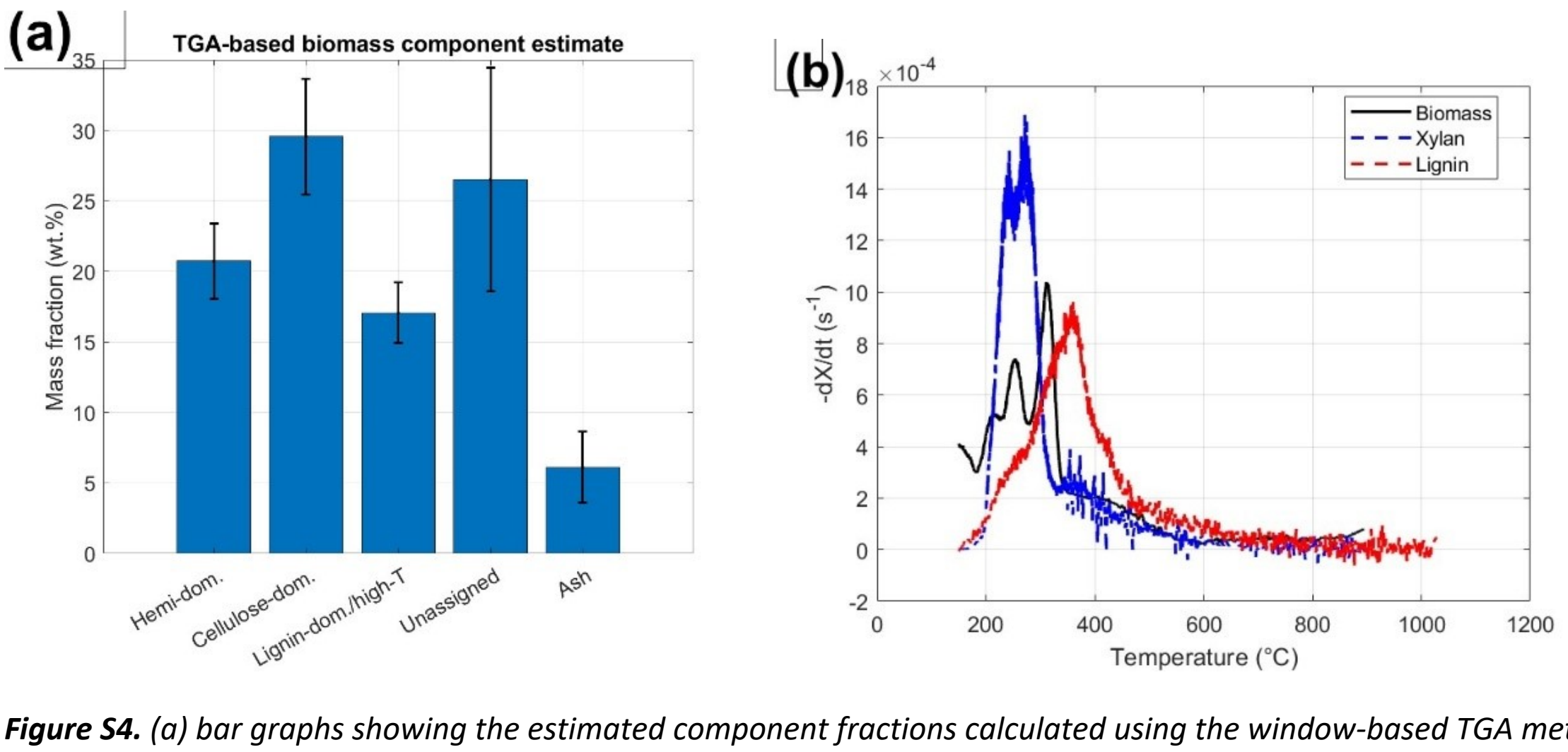

Figure S4. *(a) bar graphs showing the estimated component fractions calculated using the window-based TGA method. (b) DTG curve of Biomass, Xylan, and Lignin, showcasing area if significant signal overlap.*

As shown in Figure S4, the window-based TGA method estimated kale stem biomass into 20.69 ± 2.73 wt.% hemicellulose-dominated fraction, 29.61 ± 4.09 wt.% cellulose-dominated fraction, 17.06 ± 2.15 wt.% lignin-dominated/high-temperature fraction, 26.52 ± 7.96 wt.% unassigned organic fraction, and 6.12 ± 2.50 wt.% ash on a dry basis. Literature values reported for related herbaceous lignocellulosic biomass can range from 20-50 wt.% for cellulose, 15–40% for hemicellulose, and 5–20 wt.% for lignin, respectively. [10,11] Relative to these values, the present lignin-dominated/high-temperature fraction and ash content are broadly plausible, whereas the cellulose-dominated fraction is lower than expected for many herbaceous materials. The large, unassigned fraction indicates substantial overlap of components and supports interpreting the window-method outputs as TGA-derived fractions rather than compositional values.

Because specific compositional data for kale stem are limited, the comparison was made primarily against general herbaceous lignocellulosic literature, and should therefore be interpreted as contextual rather than exact values.

3. Particle size determination

Table S5. *Calculated Feret size and average size of particles analysed from SEM images of the Cu-activated samples.*

Sample	Feret/ μm	Average size/ μm^2
Cu5-900-2	0.32	0.135
Cu20-900-2	0.94	1.13
C40-900-2	1.25	1.77
Cu80-900-2	1.89	2.58
Cu100-900-2	2.05	3.12
Cu150-900-2	3.68	13.9
Cu350-900-2	5.69	38.3

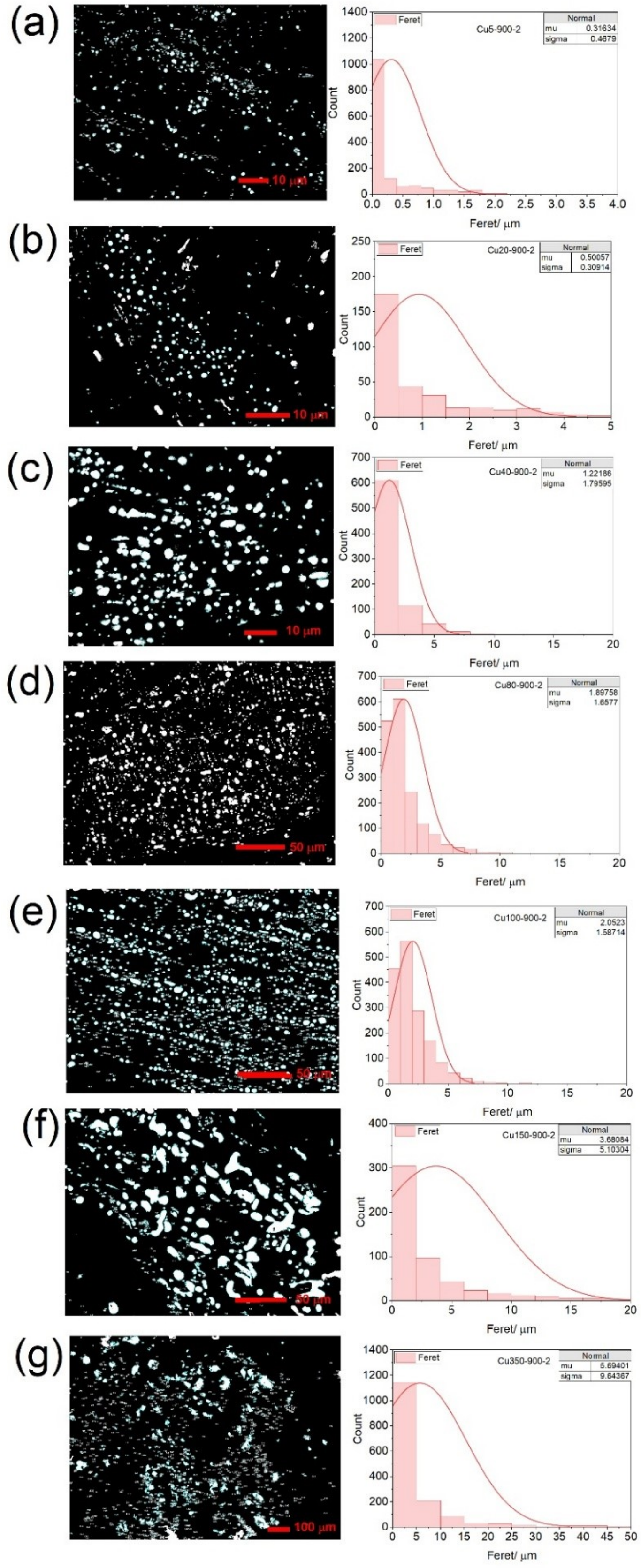

Figure S5. *Representative SEM micrographs showing the particle size distribution of Cu-activated samples carbonised at 900°C, panels (a-g) correspond to samples, Cu5-900-2, Cu20-900-2, C40-900-2, Cu80-900-2, Cu100-900-2, Cu150-900-2, and Cu350-900-2, respectively. the right column presents the corresponding Feret diameter distributions obtained from image analysis.*

4. Powder X-ray diffraction data

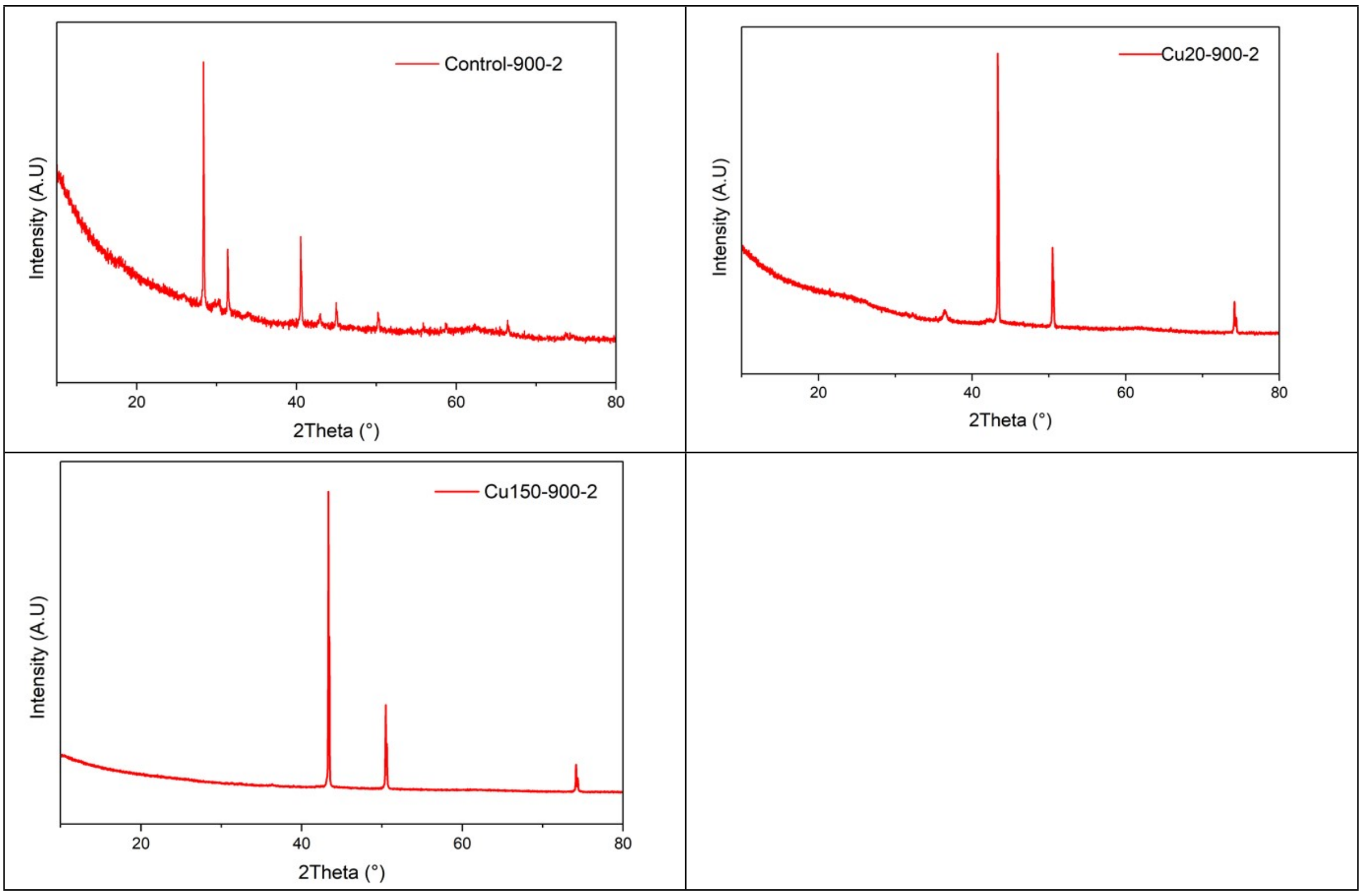

Figure S6*. PXRD patterns of (a) Control-900-2, (b) Cu20-900-2, and (c) Cu150-900-2 activated carbon samples.*

5. Elemental analysis and TEM imaging

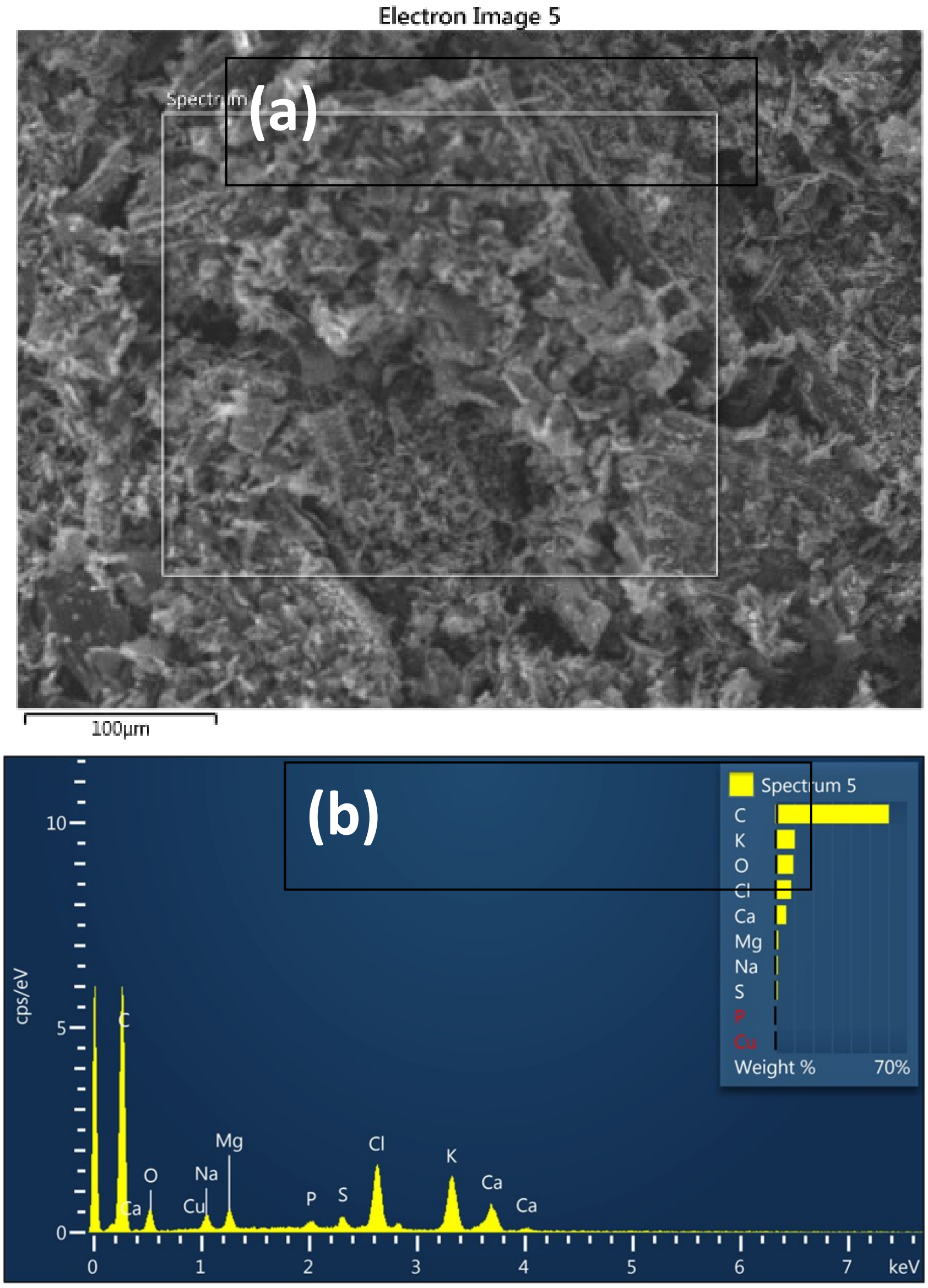

Figure S7. *(a) SEM and (b) EDX elemental analysis for the control sample*

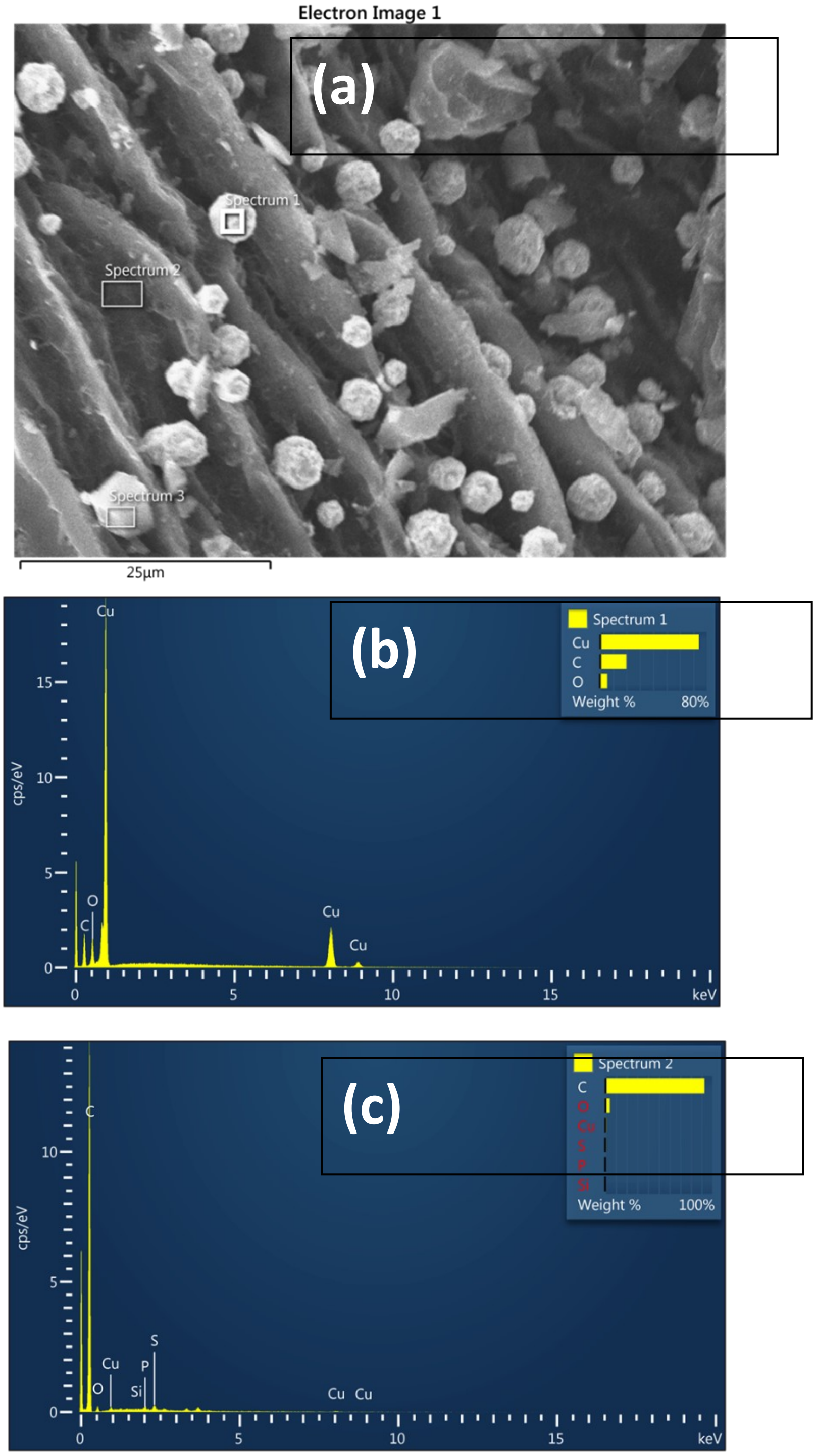

Figure S8. *(a) SEM and (b, c) EDX elemental scan of spectra 1 and 2 for the Cu20-900-2 sample, respectively.*

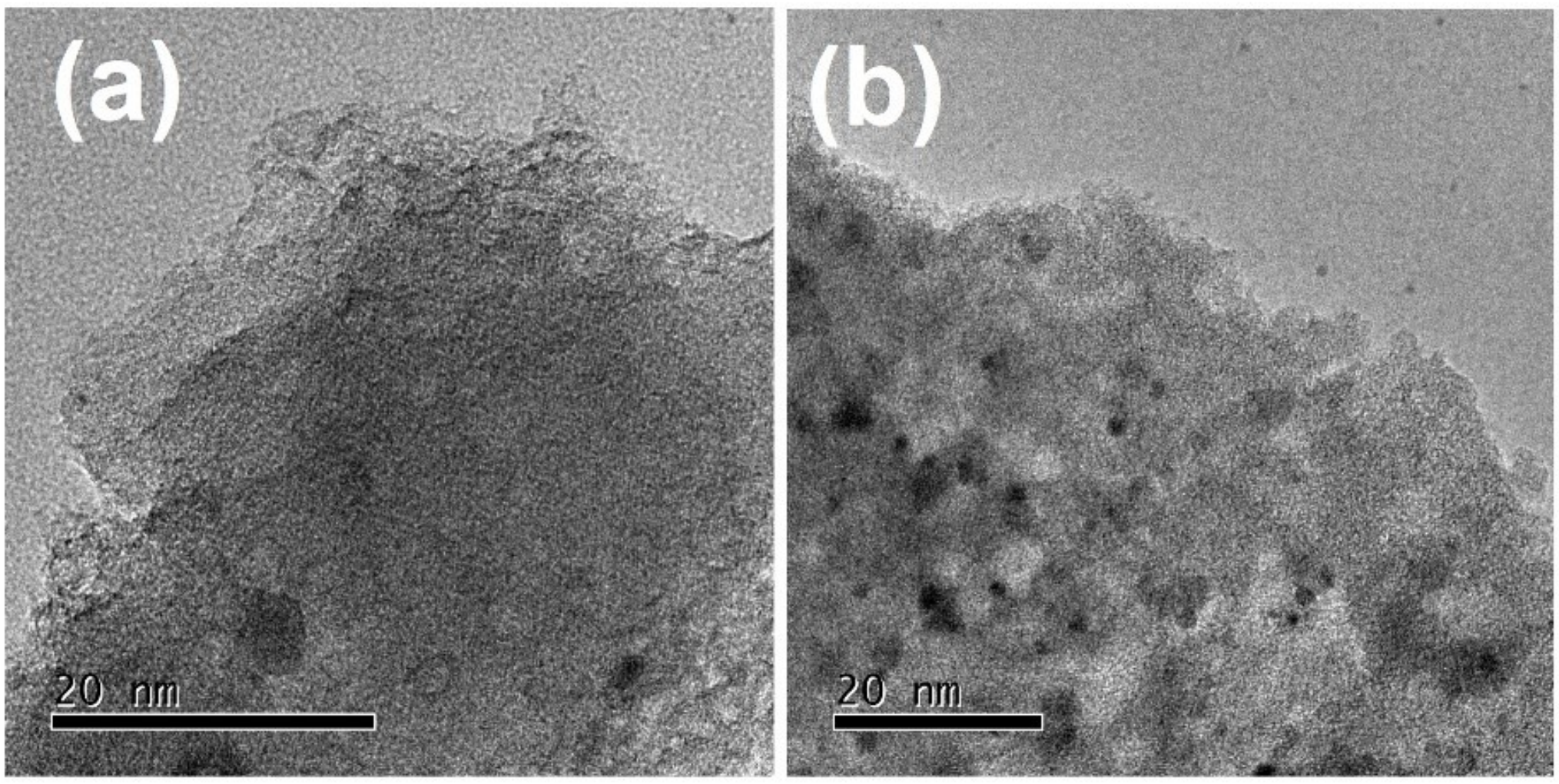

Figure S9*. TEM images of (a) Cu40-900-2, and (b) Cu80-900-2.*

6. Raman spectroscopy

Table S6. Raman deconvoluted spectra for activated carbon samples, in wavenumber ranges of 1100 to 1800 cm^{-1}.

**Control-900-2**	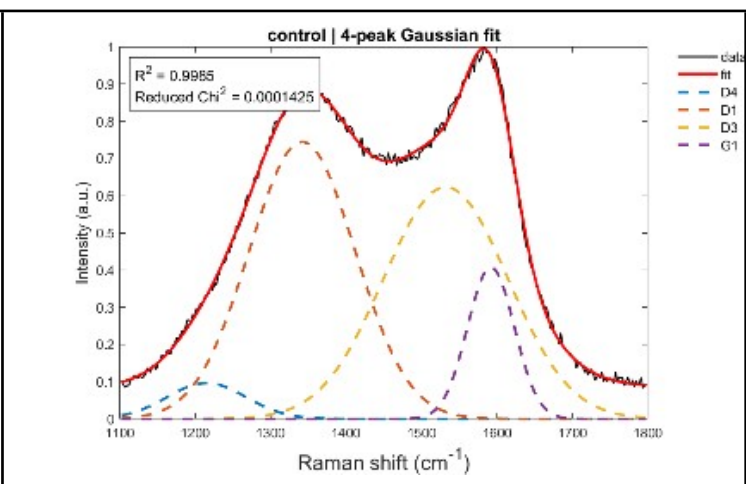	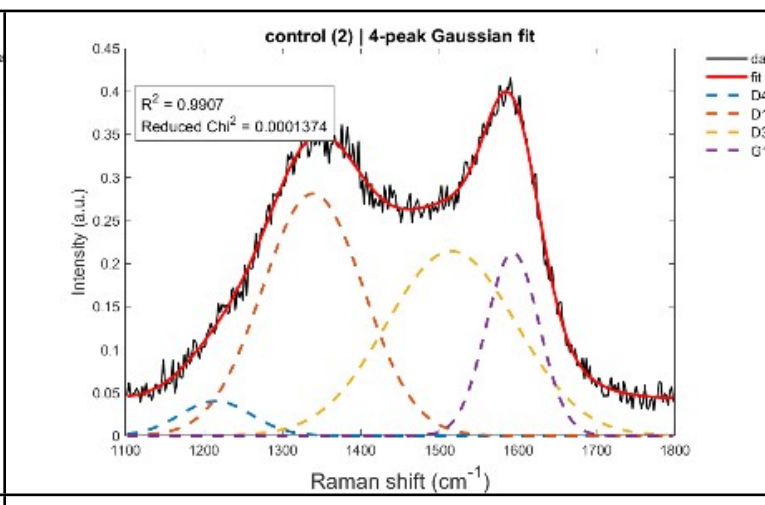	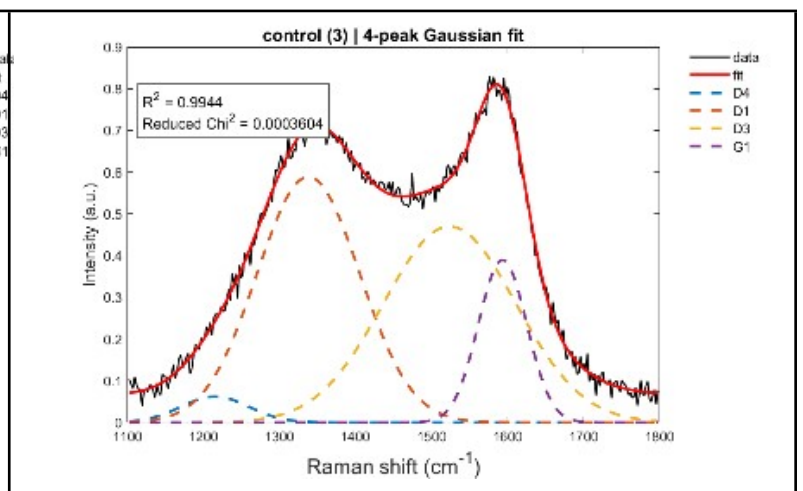
**Cu5-900-2**	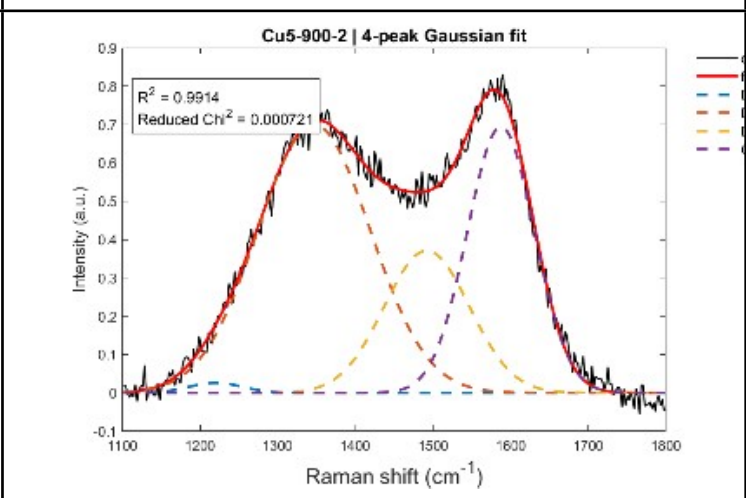	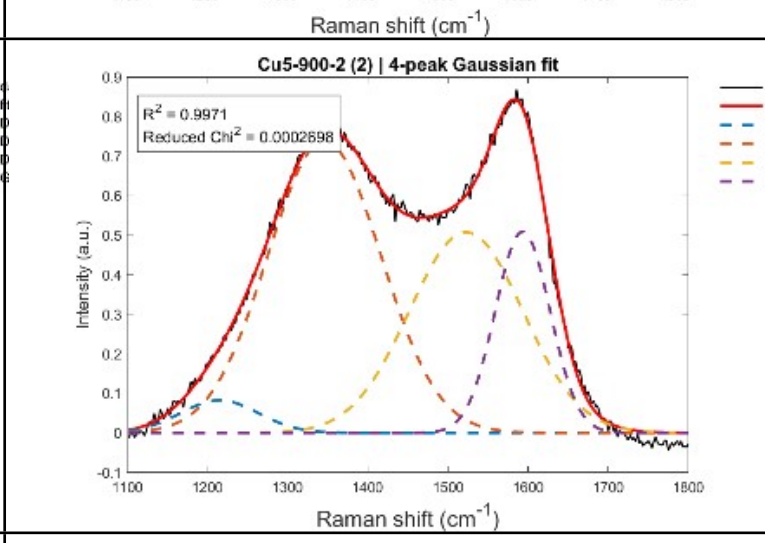	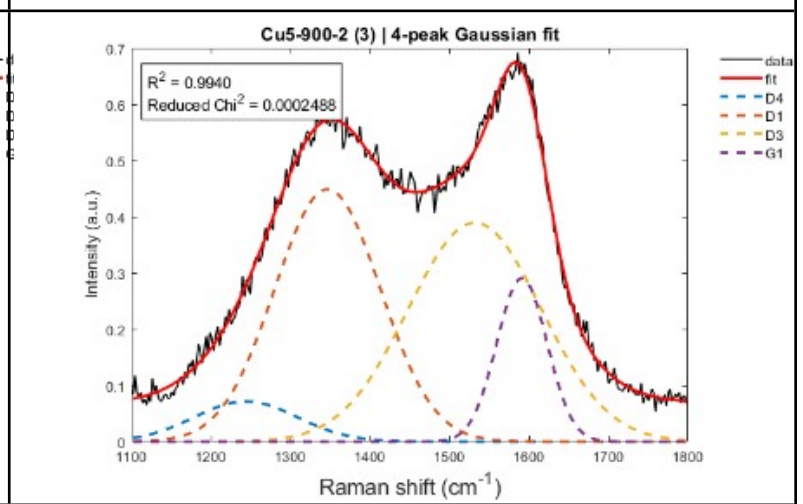
**Cu20-900-2**	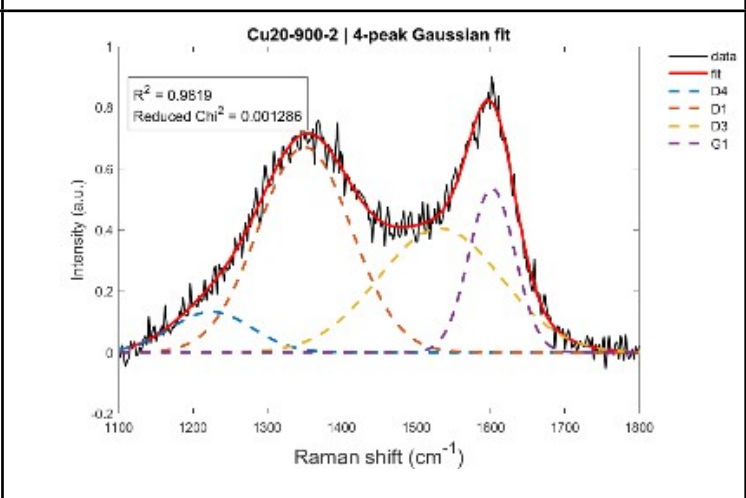	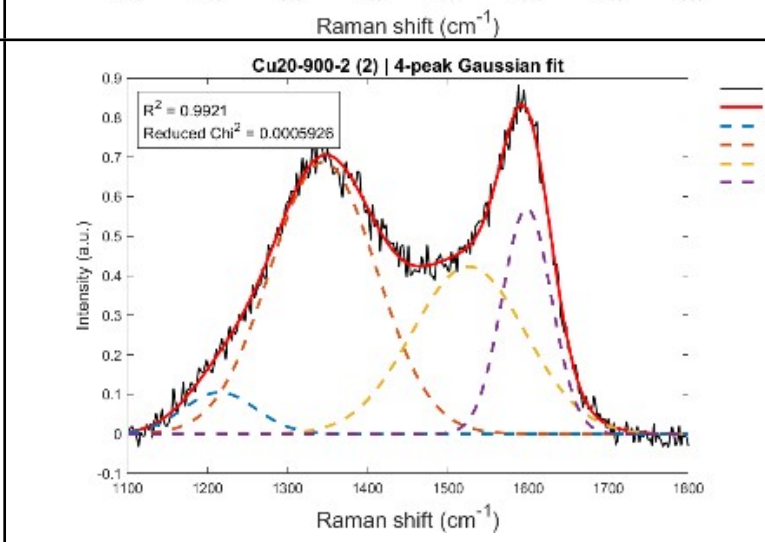	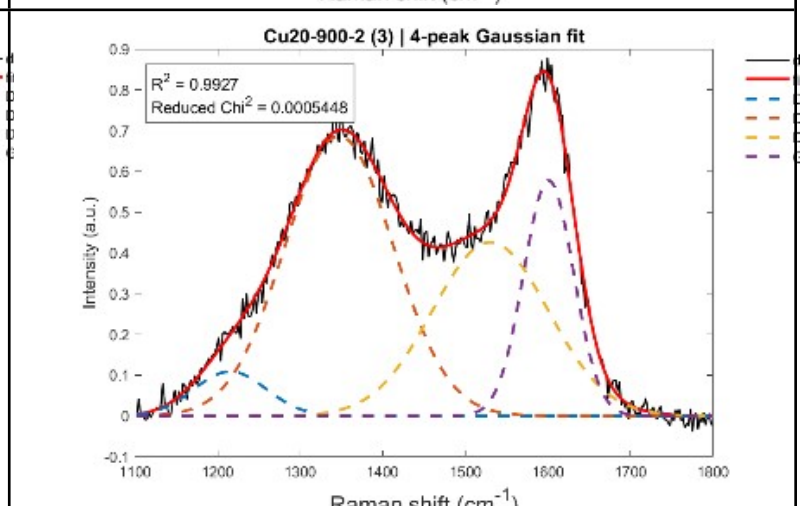
**Cu40-900-2**	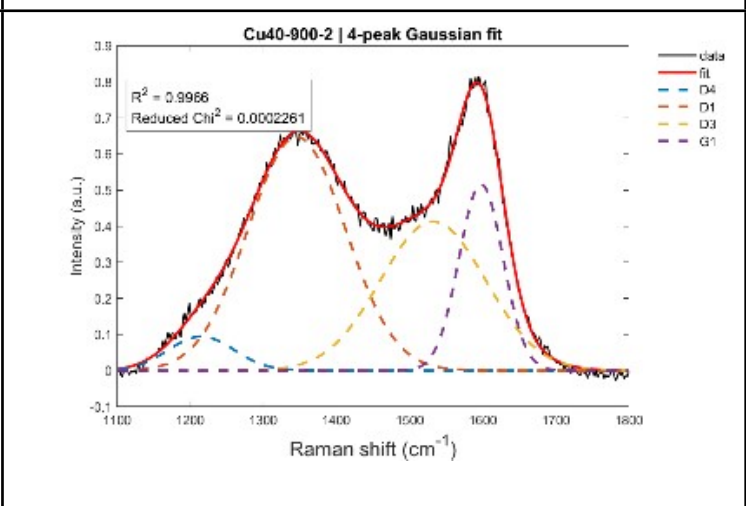	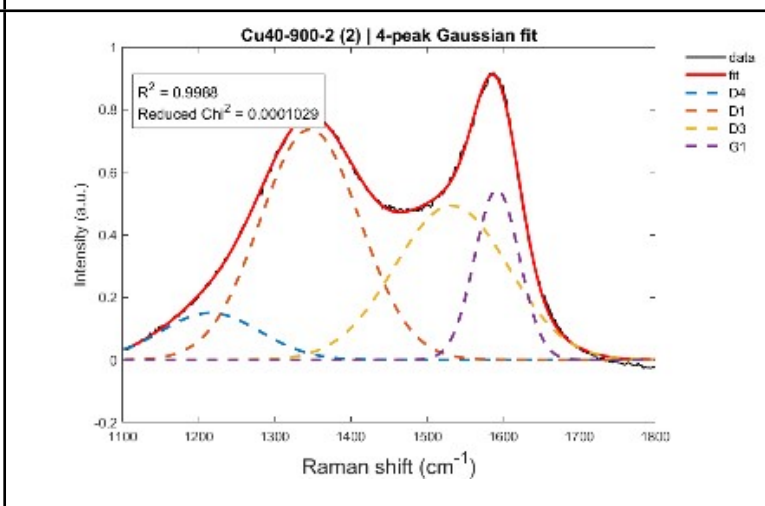	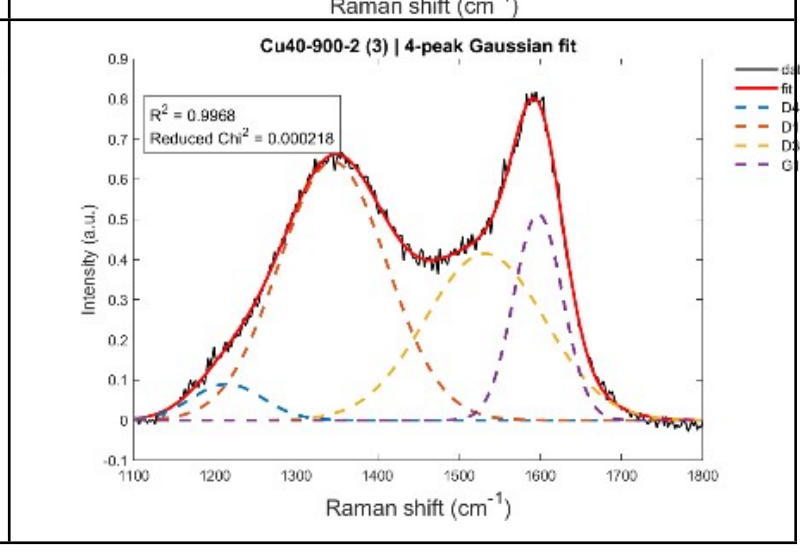

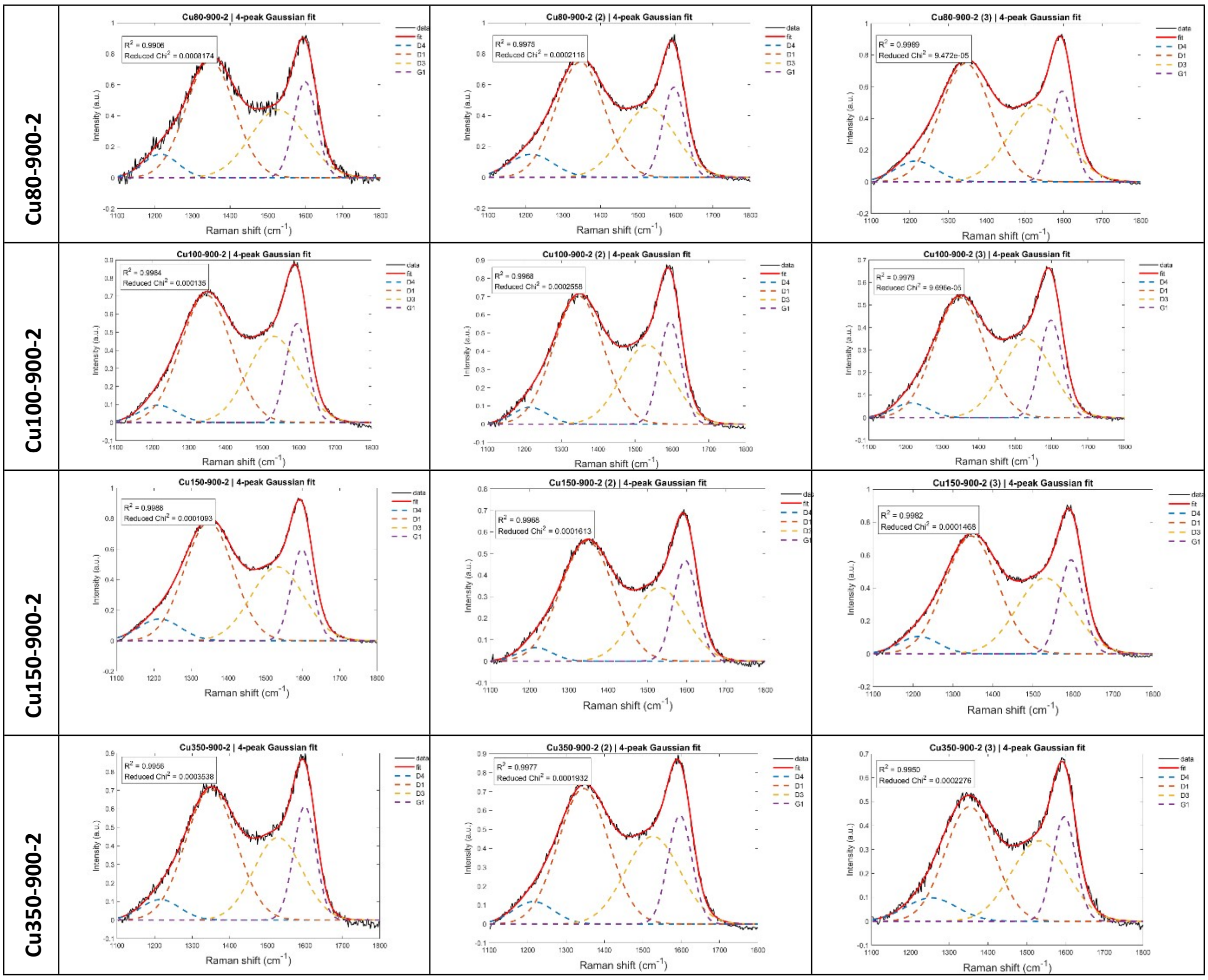

Table S7. *Table showing the mean ID1/IG ratios as well as the mean FWHM*

Sample	D1 (xc_2) ±SE (cm^{-1})	FWHM $_{(D1)}$±SE	G1 (±SE) (cm^{-1})	FWHM $_{(G1)}$±SE
Control-900-2	1339.47±1.14	158.83±3.93	1592.87±0.49	75.77±1.81
Cu5-900-2	1346.44±0.33	164.61±4.31	1590.62±1.69	85.67±8.35
Cu20-900-2	1347.30±1.72	152.66±2.81	1600.43±1.08	72.07±0.29
Cu40-900-2	1345.63±0.35	155.83±0.77	1595.65±1.92	70.13±0.49
Cu80-900-2	1345.95±0.68	153.01±1.56	1596.91±1.28	69.13±1.24
Cu100-900-2	1347.37±0.36	162.15±1.50	1596.51±0.90	71.52±0.75
Cu150-900-2	1346.73±0.63	156.68±1.53	1596.51±0.67	69.92±0.93
Cu350-900-2	1349.55±1.91	154.22±3.15	1597.90±1.06	71.07±1.04

7. BET analysis

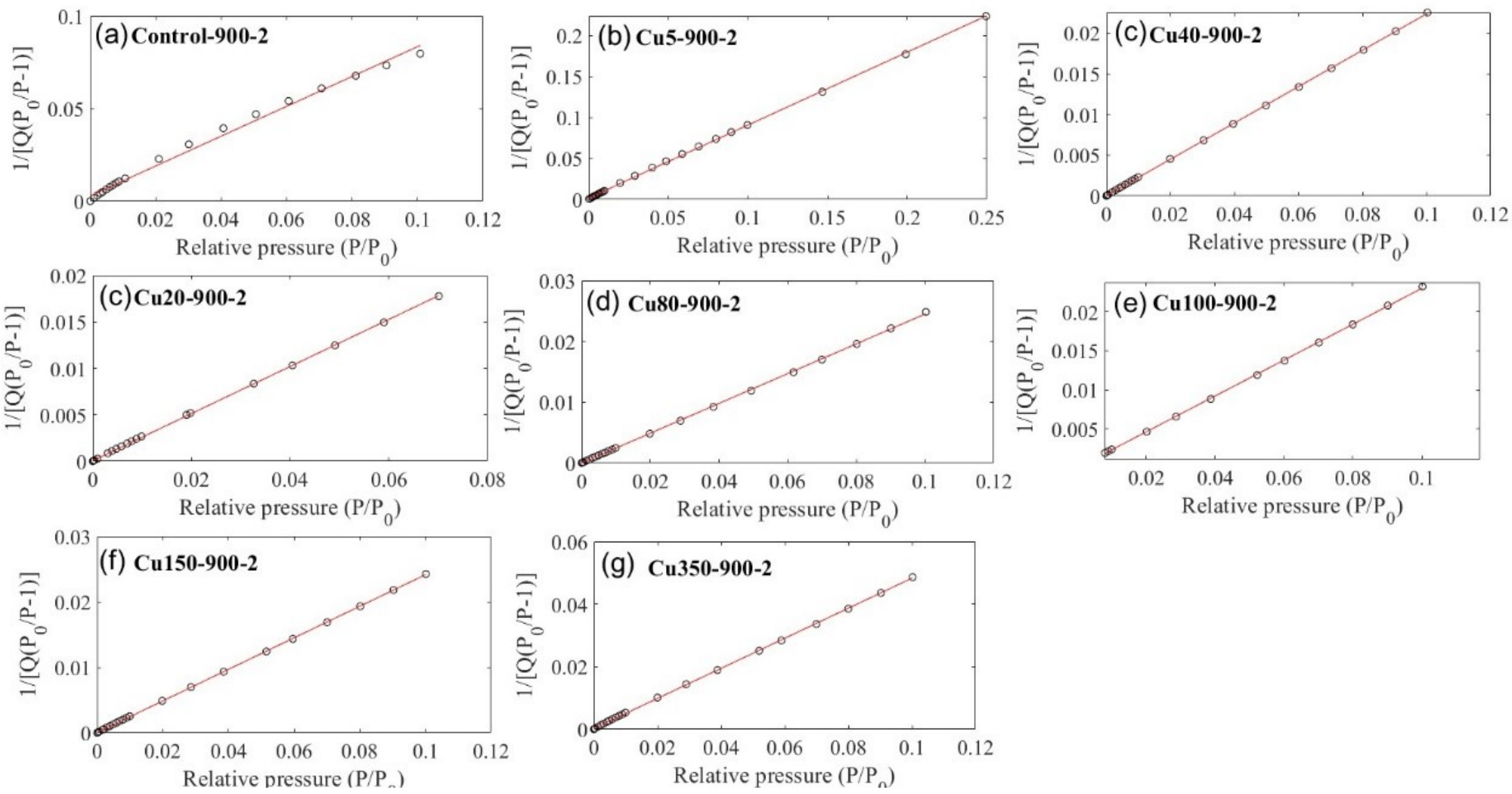

Figure S10. *table showing BET surface area plots for activated carbon samples investigated.*

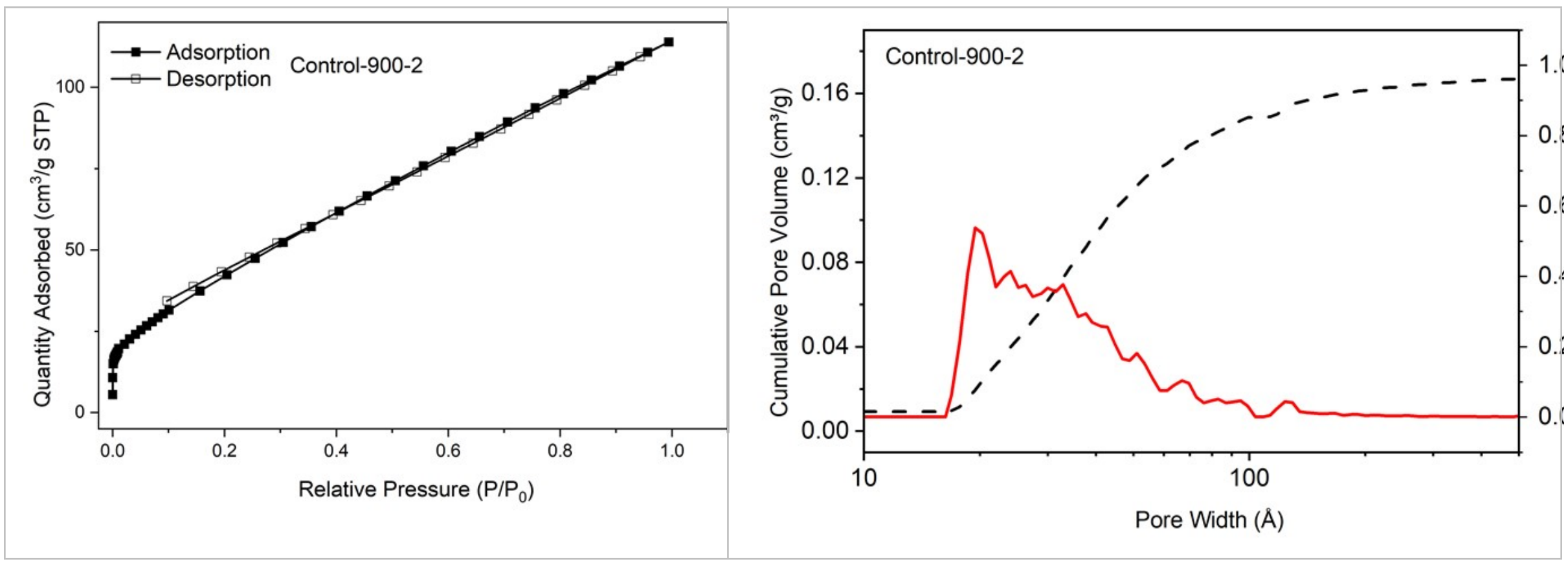

Figure S11. *N_2 sorption isotherm and pore size distribution for control sample.*

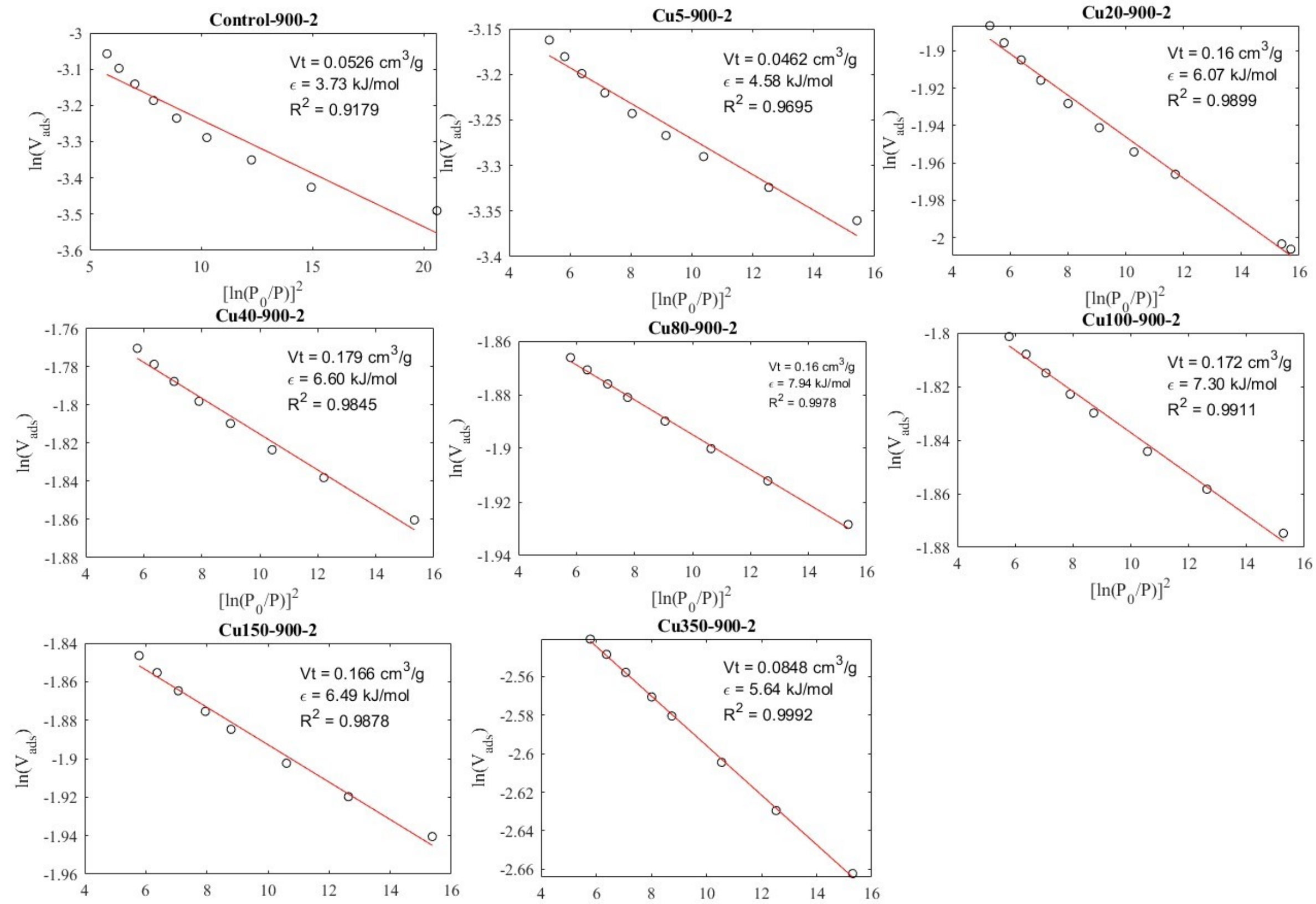

Figure S12. *Dubinin–Radushkevich plots for activated carbon samples*

Table S8. *Comparison of BET surface area, H_2 uptake at 77 K and 1 bar, and normalised uptake values for literature-reported activated carbons and $CuCl_2$-activated samples from this work a normalised uptake calculated as (H_2 uptake / BET area) × 1000 $m^2\ g^{-1}$.*

**Material**	**Activation method**	**BET surface area (m^2g^{-1})**	**H_2 uptake At 1 bar and 77K (wt%)**	**Normalised uptake at 1 bar and 77 K (wt% per 1000 m^2) [a]**	**Ref.**
Activated carbon (olive stone)	KOH (one-step)	1269	2.71	2.14	[12]
Activated carbon (Cellulose acetate derived)	KOH (one-step)	3771	3.90	1.03	[13]
Activated carbon (Chitosan derived)	KOH (one-step)	2919	2.71	0.93	[14]
Activated carbon (Hemp stem)	KOH (one-step)	922	1.57	1.7	[15]
Cu20-900-2	$CuCl_2$ (one-step)	386	0.98	2.54	This work
Cu40-900-2	$CuCl_2$ (one-step)	438	0.91	2.06	This work
Cu80-900-2	$CuCl_2$ (one-step)	408	0.83	2.03	This work
Cu100-900-2	$CuCl_2$ (one-step)	431	0.82	1.82	This work

[a] Normalised uptake calculated by dividing hydrogen uptake (wt%) by BET surface area and scaling to 1000 $m^2\ g^{-1}$

References

1 S. B. Singh and M. De, *Materials Chemistry and Physics*, 2020, **239**, 122102.
2 S. B. Singh and M. De, *International Journal of Energy Research*, 2019, **43**, 4264–4280.
3 N. P. Stadie, J. J. Vajo, R. W. Cumberland, A. A. Wilson, C. C. Ahn and B. Fultz, *Langmuir*, 2012, **28**, 10057–10063.
4 L. Naheed, M. Koppel, M. Paalo, K. Alsabawi, K. E. Lamb, E. MacA. Gray, A. Jänes and C. J. Webb, *International Journal of Hydrogen Energy*, 2021, **46**, 15761–15772.
5 H. S. Kim, J. P. Singer, Y. Gogotsi and J. E. Fischer, *Microporous and Mesoporous Materials*, 2009, **120**, 267–271.
6 G. Conte, A. Policicchio, M. Idrees, G. Desiderio and R. G. Agostino, *International Journal of Hydrogen Energy*, 2024, **50**, 763–773.
7 M. Sevilla and A. B. Fuertes, *Carbon*, 2009, **47**, 2281–2289.
8 S. Beak, S. Kim, S. Oh and J. Bae, *Journal of Environmental Chemical Engineering*, 2025, **13**, 116086.
9 X. Liu, C. Zhang, Z. Geng and M. Cai, *Microporous and Mesoporous Materials*, 2014, **194**, 60–65.
10 A. Langsdorf, M. Volkmar, D. Holtmann and R. Ulber, *Bioresour. Bioprocess.*, 2021, **8**, 19.
11 N. Reddy and Y. Yang, *Trends in Biotechnology*, 2005, **23**, 22–27.
12 N. Bader, R. Zacharia, O. Abdelmottaleb and D. Cossement, *J Porous Mater*, 2018, **25**, 221–234.
13 L. S. Blankenship, N. Balahmar and R. Mokaya, *Nat Commun*, 2017, **8**, 1545.
14 I. Wróbel-Iwaniec, N. Díez and G. Gryglewicz, *International Journal of Hydrogen Energy*, 2015, **40**, 5788–5796.
15 S. Zhang, L. H. Lee, Y. Sun and Y. Liu, *IOP Conf. Ser.: Earth Environ. Sci.*, 2021, **632**, 052087.
